# Supplementary material for: Non‐canonical cMet regulation by vimentin mediates Plk1 inhibitor–induced apoptosis
Source: EMBO Mol Med. 2019 Apr 30;11(5):e9960. doi: 10.15252/emmm.201809960 (PMC6505578; doi:10.15252/emmm.201809960)
Supplement: Supplementary file 1 — Appendix [file EMMM-11-e9960-s001.pdf]

Noncanonical cMet activation mediates resistance to Plk1 inhibitor–induced apoptosis

Ratnakar Singh[1,5], Shaohua Peng[1], Pavitra Viswanath[1,4,5], Vaishnavi Sambandam[1], Li Shen[2], Xiayu Rao[2], Bingliang Fang[3], Jing Wang[2,4], Faye M. Johnson[1,4]

APPENDIX

- **Appendix figure legends**
- ***Appendix Figure S1:* Non-small cell lung cancer cell lines used in different datasets.**
- ***Appendix Figure S2:* Changes in protein expression following TGF-β incubation in non-small cell lung cancer cell lines.**
- ***Appendix Figure S3:* TGF-β–induced isogenic cell lines are more sensitive to Plk1 inhibition than are parental lines.**
- ***Appendix Figure S4:* Co-targeting of Plk1 and cMet decreases colony formation ability.**
- ***Appendix Figure S5:* cMet inhibition increases the sensitivity of the volasertib acquired resistance (VAR) cell line to Plk1 inhibition.**
- ***Appendix Table S1:* Non-small cell lung cancer cell lines and antibodies used in different datasets.**
- ***Appendix Table S2:* Half-maximal inhibitory concentration (IC_{50}) and combination index values for volasertib and tepotinib in non-small cell lung cancer cell lines.**
- ***Appendix Table S3:* Antibodies used for immunoblotting and immunoprecipitation.**
- ***Appendix Table S4:* Oligos used for real time PCR**
- ***Appendix Table S5:* Summery of statistical test and p value**

Appendix Figure S1. Non-small cell lung cancer cell lines used in different datasets. The Venn diagram shows the number of overlapping cell lines among the Cancer Therapeutics Response Portal v2 (CTRPv2) drug sensitivity database, the MD Anderson Cell Line Project (MCLP) protein expression dataset, and our prior study (CCR) (10).

Appendix Figure S2. Changes in protein expression following TGF-β incubation in non-small cell lung cancer cell lines. H1975, HCC4006, and HCC366 non-small cell lung cancer cells were treated with 5 ng/ml TGF-β for 14 days. Cells were then harvested, and lysates were analyzed for protein/phosphoprotein expression by reverse phase protein array. Experiments were performed in triplicate on three different days. Arrows indicate a decrease (red) or increase (blue) in protein expression at the end of treatment with TGF-β. For this analysis, a false discovery rate (FDR) cutoff of 0.01 was set. Several proteins known to be involved in epithelial-to-mesenchymal transition (blue text) changed expression following TGF-β incubation.

Appendix Figure S3. TGF-β–induced isogenic cell lines are more sensitive to Plk1 inhibition than are parental lines. (A) Parental and TGF-β isogenic cell lines were treated with 50nM volasertib for 72 hours. Cells were then harvested, and lysates were immunoblotted for the indicated proteins. **(B)** Cell viability of parental and TGF-β–induced mesenchymal H1975, HCC4006, and HCC366 cell lines treated with the indicated concentrations of volasertib for 72 hours was measured using CellTiter-Glo. Experiments were performed in triplicate and error bars represent standard deviation.

Appendix Figure S4. Co-targeting of Plk1 and cMet decreases colony formation ability. Shown are representative pictures of H1975, HCC366, H1792, and Calu6 cells treated as indicated with 25nM volasertib, 400nM tepotinib, both, or vehicle control for 24 hours and allowed to grow in drug-free media for 15-20 days to form colonies that were then stained with crystal violet and photographed.

Appendix Figure S5. cMet inhibition increases the sensitivity of the volasertib acquired resistance (VAR) cell line to Plk1 inhibition. (A) Shown are representative pictures of Calu6 parental and Calu6-VAR cells treated as indicated for 24 hours and allowed to grow in drug-free medium for 15-20 days to form colonies, which were then stained with crystal violet and photographed. **(B)** Cell viability of Calu6 parental and VAR cells treated with the indicated concentrations of volasertib for 72 hours was measured using CellTiter-Glo. Experiments were performed in triplicate and error bars represent standard deviation.

Appendix Figure S1

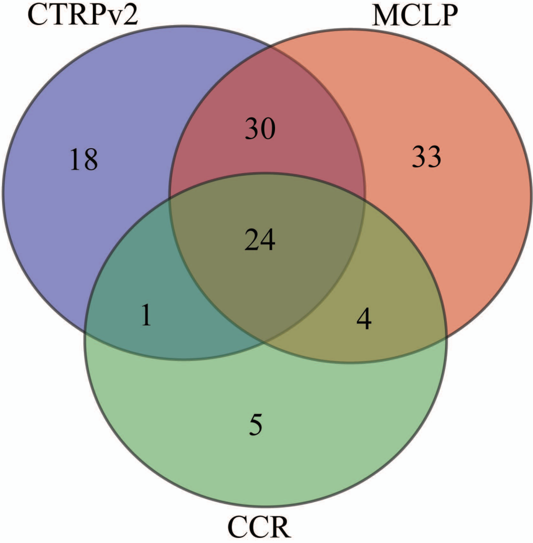

Appendix Figure S2

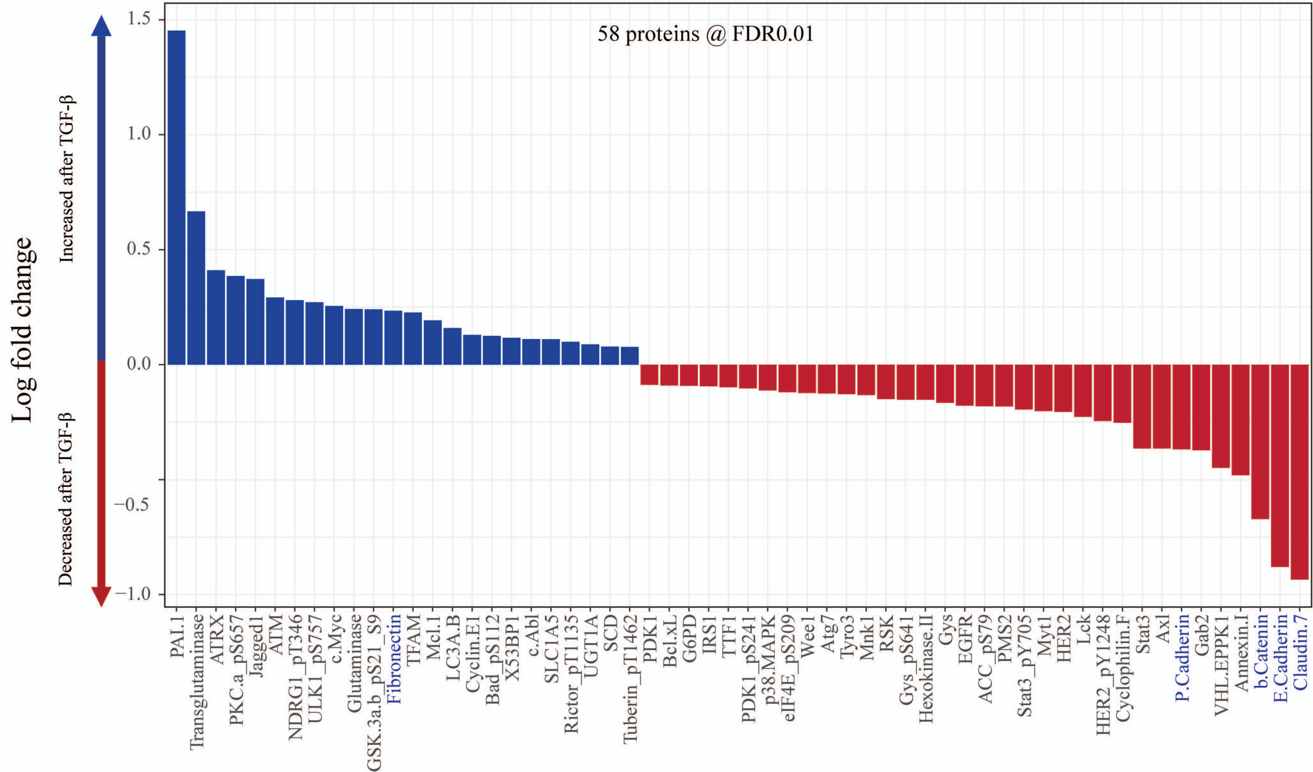

Appendix Figure S3

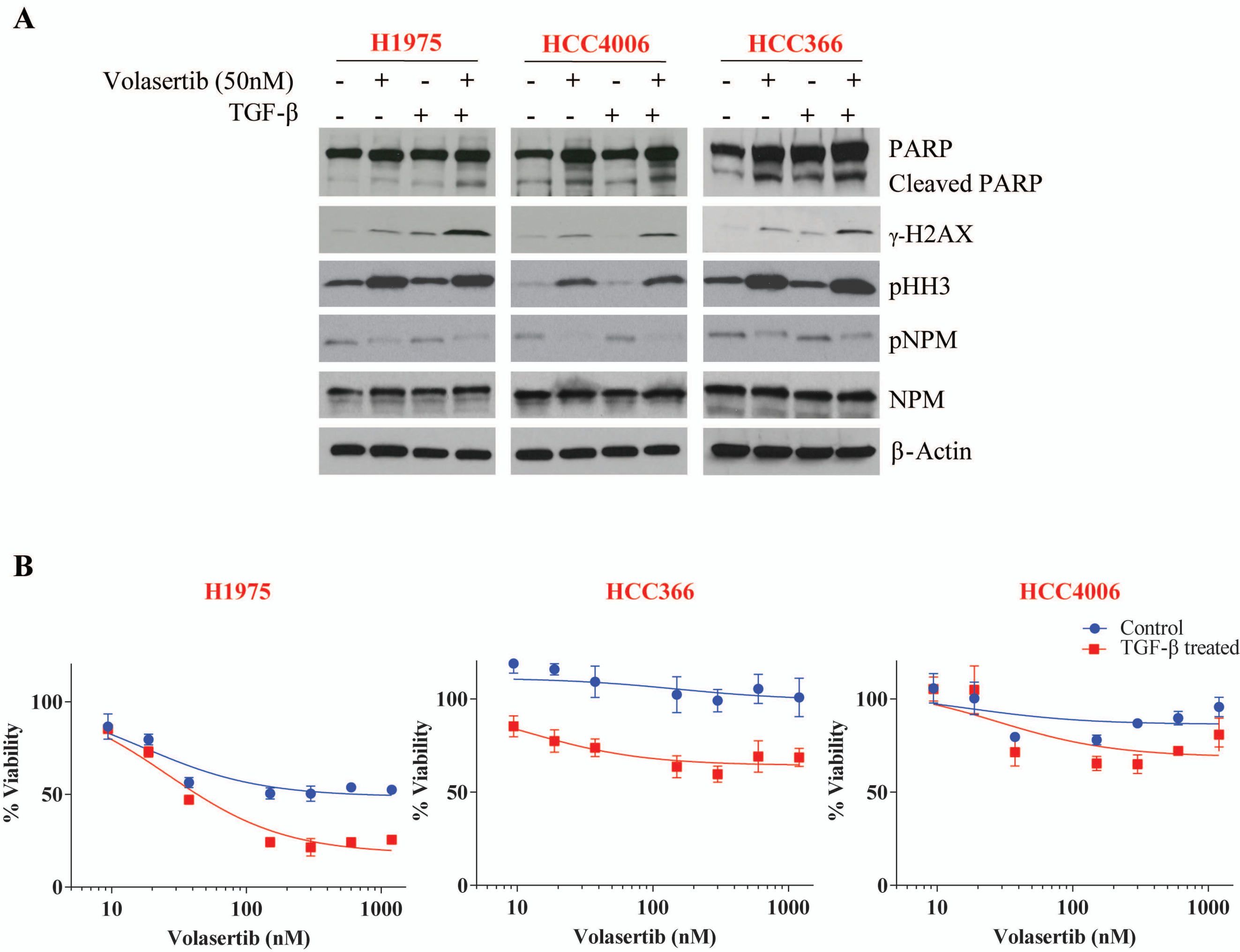

Appendix Figure S4

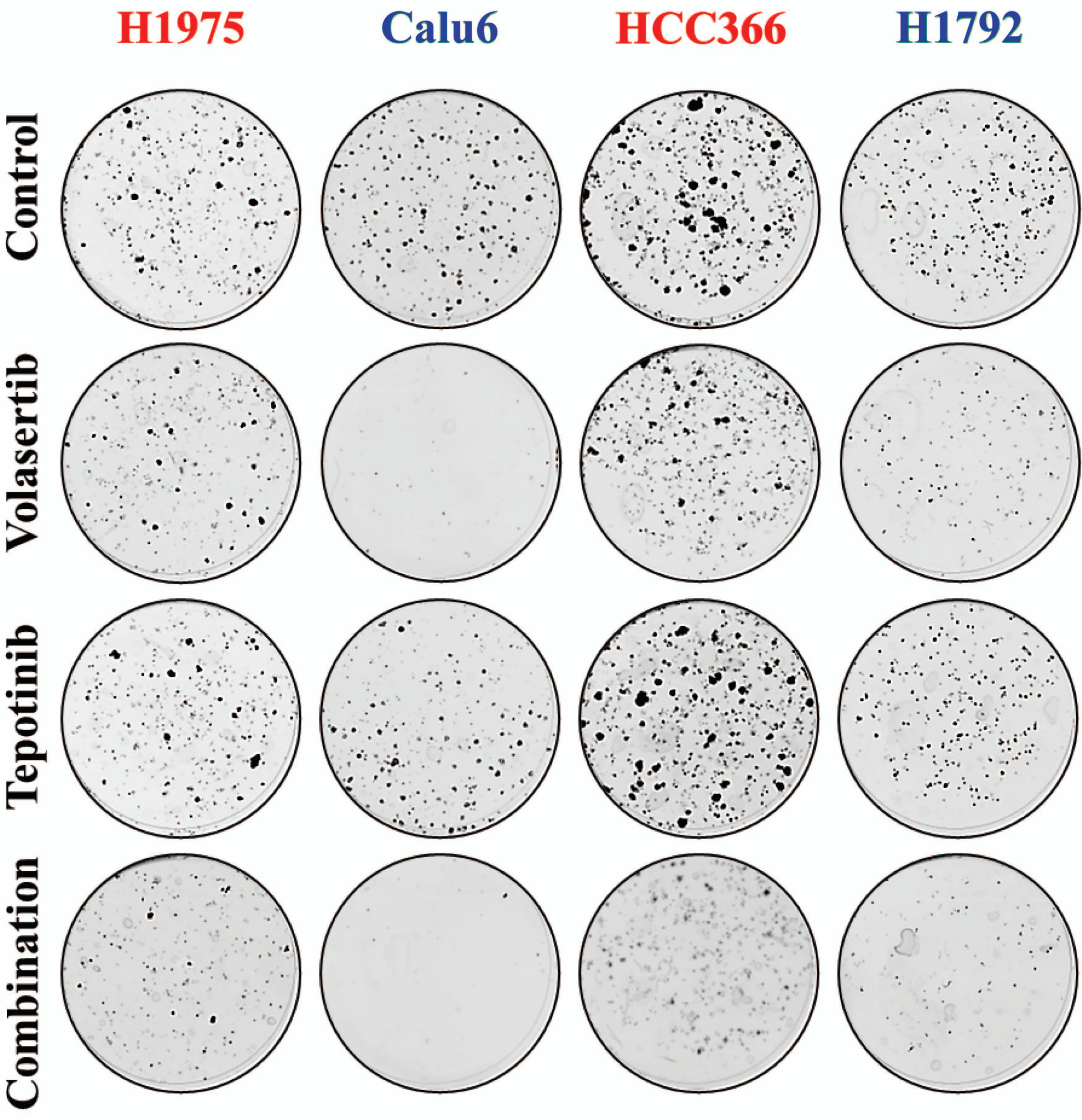

Appendix Figure S5

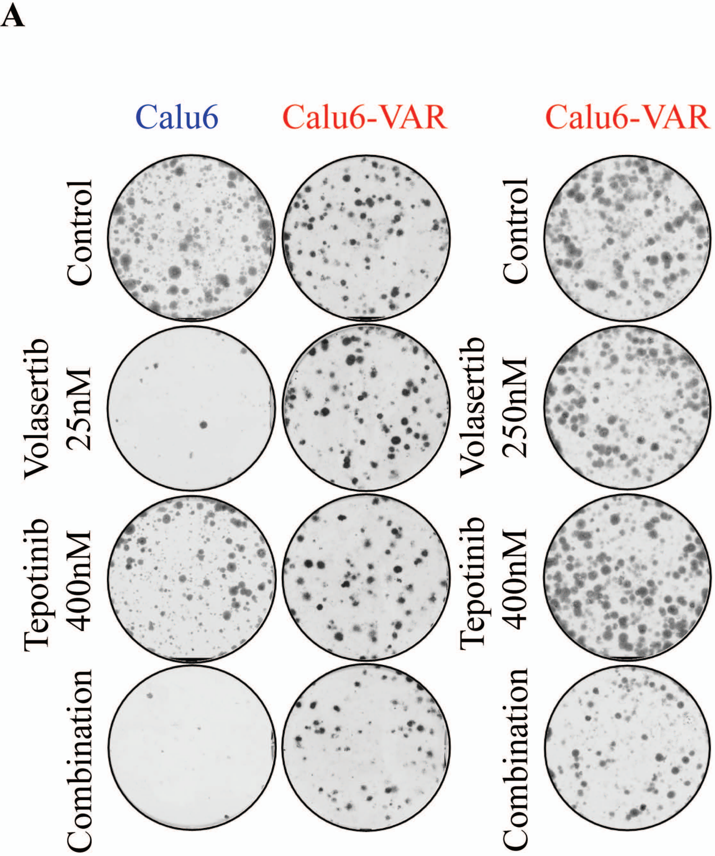

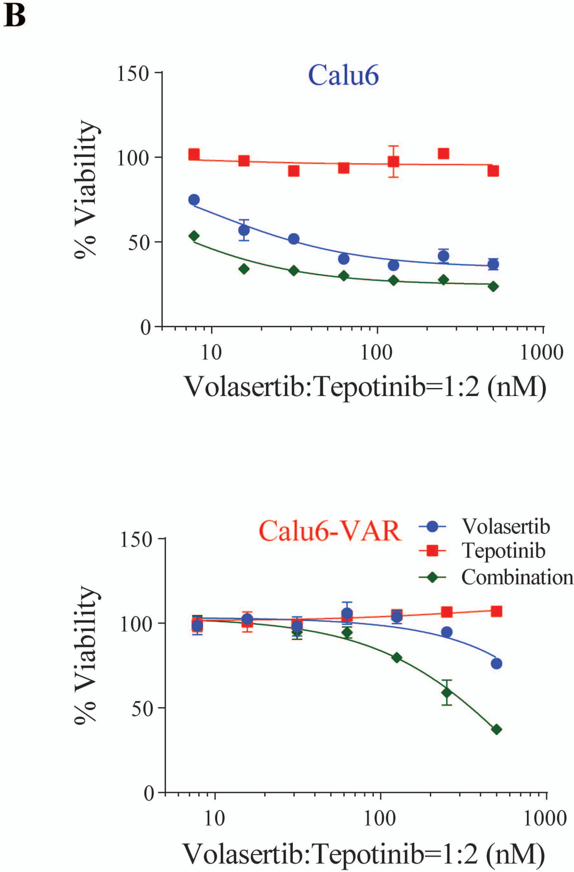

Appendix Table S1: Non-small cell lung cancer cell lines and antibodies used in different datasets.

Present study							Previous study (reference 22)					
CTRPv2 screening (no. cell lines/total cell lines)							Drug screening (no. cell lines/total cell lines)					
BI2536 (53/71)	GSK461364 (51/69)	BRDK70511574 (49/68)	GW843682X (48/64)	All four drugs (45/60)	MCLP RPPA (91)	Cell lines in both CTRPv2 and MCLP RPPA (45)	Volasertib (34/50)	GSK461364 (42/72)	BI2536 (34/50)	All three Drugs (34/50)	RPPA (45)	Cell lines in both drug screening and RPPA (34)
H1563	H1385	H1385	H1563	H460	A427	CALU3	CALU-6	CALU-6	CALU-6	CALU-6	CALU6	H1437
H1573	H1563	H1563	H1573	CALU3	A549	H460	H1703	H1703	H1703	H1703	H23	HCC4006
H1734	H1573	H1573	H1869	H2286	CALU3	SKLU1	H1155	H1155	H1155	H1155	H1792	H460
H1869	H1869	H1734	H1944	CALU1	CALU6	H1299	H2087	H2087	H2087	H2087	H226	H520
H1944	H1944	H1869	H2126	H524	DFCI024	H1650	H460	H460	H460	H460	HCC461	H23
H2126	H2126	H1944	H2172	SKLU1	DFCI032	H1792	H838	H838	H838	H838	A549	H322
H2172	H2172	H2126	H650	H1299	EKVX	H1703	H1299	H1299	H1299	H1299	H460	H2009
H650	H650	H2172	H727	H1650	H1155	HCC1195	H1355	H1355	H1355	H1355	CALU1	H1155
H727	H727	H650	HCC1195	H1623	H125	H2170	H1792	H1792	H1792	H1792	H1563	HCC366
HCC1195	HCC1195	H727	HCC2108	H1792	H1299	HCC2108	H1944	H1944	H1944	H1944	H838	H1993
HCC2108	HCC2108	HCC1195	H1155	H1703	H1355	H1666	H1666	H1666	H1666	H1666	H1355	H1299
SKMES1	SKMES1	HCC2108	H2030	HCC1195	H1373	H1395	H650	H650	H650	H650	HCC4017	H1650
SW1573	H358	SKMES1	H2228	H2170	H1385	H1355	H441	H441	H441	H441	HCC44	H1792
H358	H1155	H358	CALU6	HCC2108	H1395	H1793	HCC4017	HCC4017	HCC4017	HCC4017	H1155	H1703
H1155	H2030	H1155	H1299	H1666	H1437	H1373	H23	H23	H23	H23	H1299	H2170
H2030	H2228	H2030	H1373	H1395	H1563	H2030	H1838	H1838	H1838	H1838	H1703	H838
H2228	CALU6	H2228	H1395	H1355	H1568	HCC827	HCC461	HCC461	HCC461	HCC461	HCC15	H1666
CALU6	H1299	CALU6	H1651	H1793	H1573	CALU6	CALU-1	CALU-1	CALU-1	CALU-1	H1373	HCC4017
H1299	H1373	H1299	H1838	H1373	H1648	H2228	H1793	H1793	H1793	H1793	HCC366	H1355
H1373	H1395	H1373	H2087	H2030	H1650	H2023	SK-LU-1	SK-LU-1	SK-LU-1	SK-LU-1	H1793	H1793
H1395	H1838	H1395	H292	HCC827	H1651	H661	H226	H226	H226	H226	H1975	A549
H1651	H2087	H1838	H520	CALU6	H1666	HCC15	HOP-62	HOP-62	HOP-62	HOP-62	H520	H1373
H1838	H292	H2087	H661	H2228	H1693	H2085	H1373	H1373	H1373	H1373	H1650	H226
H2087	H520	H292	HCC15	H2023	H1703	HCC95	A549	A549	A549	A549	H9944	H1648
H292	H661	H520	H2342	H661	H1770	H1838	H1395	H1395	H1395	H1395	H1666	HCC44
H520	HCC15	H661	HCC1833	HCC15	H1781	H2087	CAL12T	CAL12T	CAL12T	CAL12T	H2085	H441
H661	H1975	HCC15	SKLU1	H2085	H1792	H520	H1437	H1437	H1437	H1437	HCC95	HCC461
HCC15	H1693	H2342	H1355	HCC95	H1793	H23	H1568	H1568	H1568	H1568	H1993	HCC15
H1975	H2342	HCC1833	H1793	H1838	H1819	H1155	H157	H157	H157	H157	H441	H2122
H1693	HCC1833	SKLU1	H2170	H446	H1838	HCC2279	H2122	H2122	H2122	H2122	EKVX	H1975
H2342	SKLU1	H1355	HCC95	H2087	H1869	HCC366	H1651	H1651	H1651	H1651	H1437	H358
HCC1833	H1355	H1793	H1666	H520	H1944	H1869	H661	H661	H661	H661	H1648	HCC95
SKLU1	H1793	H2170	H2085	H23	H1975	H522	HCC1359	HCC1359	HCC1359	HCC1359	H322	H2126
H1355	H2170	HCC95	H2106	H1155	H1993	H1563	HCC193	HCC193	HCC193	HCC193	H3122	H2087
H1793	HCC95	H1666	HCC2279	H727	H2023	H650	HCC44	HCC44	HCC44	HCC44	H1819	
H2170	H1666	H2085	HCC1438	HCC2279	H2030	A549	HCC95	HCC95	HCC95	HCC95	H2009	
HCC95	H2085	H2106	HCC366	HCC366	H2073	H2342	H1648	H1648	H1648	H1648	H358	

H1666	H2106	HCC2279	A549	H69	H2077	H1573	H1650	H1650	H1650	H1650	HCC515
H2085	HCC2279	HCC1438	H23	H1869	H2085	H441	H1975	H1975	H1975	H1975	HCC4006
H2106	HCC1438	HCC366	H226	H522	H2087	H2122	H1993	H1993	H1993	H1993	H2347
HCC2279	HCC366	A549	H522	H810	H2106	H1944	H2009	H2009	H2009	H2009	H3255
HCC1438	A549	H23	H446	H1563	H2122	HCC1438	H2126	H2126	H2126	H2126	H2126
HCC366	H23	H226	H524	H650	H2126	H2126	H2170	H2170	H2170	H2170	H2087
A549	H226	H522	DMS79	H2110	H2170	HCC1833	H322	H322	H322	H322	H2122
H23	H522	H446	H211	HCC78	H2228	H2106	H358	H358	H358	H358	H2170
H226	H446	H524	H69	H1581	H2250		H520	H520	H520	H520	
H522	H524	DMS79	HCC78	A549	H2258		HCC15	HCC15	HCC15	HCC15	
H446	DMS79	H211	H2110	H2172	H23		HCC2302	HCC2302	HCC2302	HCC2302	
H524	H211	H69	H1568	H2342	H2342		HCC366	HCC366	HCC366	HCC366	
H211	H69	HCC78	H441	H226	H2347		HCC4006	HCC4006	HCC4006	HCC4006	
H69	HCC78	H2110	H838	H1573	H2882			PC-9			
HCC78	H2110	H1568	HCC827	H441	H324			A427			
H2110	H441	H441	H1703	H211	H358			DFCI032			
H1568	H838	HCC827	H2122	H2122	H441			H2030			
H441	HCC827	H1703	H2286	H1944	H460			HCC1438			
H838	H1703	H2122	H810	HCC1438	H520			HCC4018			
HCC827	H2122	H2286	H1623	H292	H522			H2258			
H1703	H2286	H810	H2023	H2126	H596			COR-L-23			
H2122	H810	H1623	H1581	HCC1833	H650			DFCI024			
H2286	H1623	H2023	CALU1	H2106	H661			EBC-1			
H810	H2023	H1435	CALU3		H820			EKVX			
H1623	H1435	H1581	H1792		H838			H125			
H2023	H1581	SHP77	H1650		H920			H1563			
H1435	SHP77	CALU1	H460		HCC1195			H1573			
H1581	CALU1	CALU3			HCC1438			H1819			
SHP77	CALU3	H1792			HCC15			H2085			
CALU1	H1792	H1650			HCC1833			H2291			
CALU3	H1650	H460			HCC1897			H2342			
H1792	H460				HCC193			H2347			
H1650					HCC2108			H3122			
H460					HCC2279			H3255			
					HCC2374			HCC515			
					HCC2429						
					HCC2450						
					HCC2814						
					HCC3051						
					HCC364						
					HCC366						
					HCC4011						
					HCC4017						
					HCC4018						
					HCC4019						
					HCC515						
					HCC827						

HCC95	
HOP62	
HOP92	
PC9	
SKLU1	
SKMES1	
SW1573	

RPPA, reverse phase protein array; MCLP, MD Anderson Cell Line Project; CTRPv2, Cancer Therapeutics Response Portal v2.

Appendix Table S2: Half-maximal inhibitory concentration (IC_{50}) and combination index values for volasertib and tepotinib in non-small cell lung cancer cell lines.

	Single-agent IC_{50}, nM		Combination IC_{50}, nM		Combination index simulations (±SD)		
**Cell line**	**Volasertib**	**Tepotinib**	**Volasertib**	**Tepotinib**	**F_a = 0.50**	**F_a = 0.25**	**Interpretation**
H1975	>500	>1000	573.7	1147.5	0	0	Highly synergistic
Calu6	13	>1000	3.5	7	0.26±0.35	0.378±2.2	Synergistic at Fa = 0.5
H1792	154.5	>1000	137	274	0	0	Highly synergistic
HCC366	>500	1088.59	789.8	1579.7	2.09±1.6	0.17±0.08	Synergistic at Fa = 0.25
HCC650	201.5	>1000	156.4	312.9	0.77±0.48	0.29±0.15	Synergistic
EKVX	293.3	>1000	326.8	653.7	1.1±2	0.36±0.39	Additive/synergistic
HCC95	139	>1000	0.2	0.4	0	0	Highly synergistic
HCC2405	>500	>1000	1740.4	3480.8	0.114	0.157	Synergistic

Appendix Table S3: Antibodies used for immunoblotting and immunoprecipitation.

Antibody name	Host species	Supplier	Catalog Number	Dilution used	Analysis
Plk1	Rabbit	CST	4513	1:1000	WB
Plk1	Mouse	Invitrogen	37-7100	1:100	IP
Plk1 T210	Rabbit	CST	9062	1:1000	WB
Cleaved PARP	Rabbit	CST	5625	1:1000	WB
PARP	Rabbit	CST	9532	1:1000	WB
Cleaved caspase 3	Rabbit	CST	9661	1:1000	WB
cMet	Mouse	CST	3127	1:1000	WB
Phosphorylated cMet (Y1234/1235)	Rabbit	CST	3077	1:1000	WB
FAK	Rabbit	CST	13009	1:1000	WB
Phosphorylated FAK (Y397)	Rabbit	CST	3283	1:1000	WB
Phosphorylated FAK(Y925)	Rabbit	CST	3284	1:1000	WB
Src	Mouse	CST	2110	1:1000	WB
Phosphorylated Src (Y416)	Rabbit	CST	2101	1:1000	WB
NPM	Rabbit	CST	3542	1:1000	WB
Phosphorylated NPM (S4)	Rabbit	CST	3520	1:1000	WB
Phosphorylated HH3 (S10)	Rabbit	CST	9701	1:1000	WB
γ-H2AX (S139)	Rabbit	CST	9718	1:1000	WB
Akt	Rabbit	CST	4691	1:1000	WB
Phosphorylated Akt (S743)	Rabbit	CST	4060	1:1000	WB

Erk	Rabbit	CST	9102	1:1000	WB
Phosphorylated Erk (T202/204)	Rabbit	CST	4370	1:1000	WB
β-actin	Mouse	Sigma Aldrich	3700	1:20000	WB
E-cadherin	Rabbit	CST	3195	1:1000	WB
Vimentin	Rabbit	CST	5741	1:1000	WB
HGF	Rabbit	CST	52445	1:2000	WB
β1-integrin	Rabbit	CST	4706	1:1000/1:100	WB/IP
Vimentin	Mouse	CST	3390	1:100	IP
Phosphorylated vimentin (S82)	Rabbit	CST	12569	1:1000	WB
Fibronectin	Rabbit	Abcam	Ab32419	1:10000	WB

WB, Western blot analysis; IP, immunoprecipitation; CST, Cell Signaling Technology

Appendix Table S4: Oligos used for real time PCR

Oligos NAME	Sequence (5'→3')
CDH1_F	TGGTGGTGATGTCCAAAAGAT
CDH1_R	TAAGAAAATCTGCAAGGTGCTG
VIM_F	AAACAGCTTTCAAGTGCCTTTC
VIM_R	TCTTGTAGGAGTGTCGGTTGTT
TWIST_F	AGAAGTCTGCGGGCTGTG
TWIST_R	CGTCTGCAGCTCCTCGTAAG
SLUG_F	TCAGCTCAGGAGCAGACAGC
SLUG_R	ATCCGGAAAGAGGAGAGAGG
SNAIL_F	CCTCCCTGTCAGATGAGGAC
SNAIL_R	GCCTCCAAGGAAGAGACTGA
ZEB1_F	CATTTTTCCTGAGGCACCTG
ZEB1_R	TGAAAATGCATCTGGTGTTCC
ACTB_F	CACCATTGGCAATGAGCGGTTC
ACTB_R	AGGTCTTTGCGGATGTCCACGT

Appendix Table S5: Summery of statistical test and p value

Figure number	Sample			p value	Test used	Significance
**Fig 2A**	**RTPCR**	H1975	CDH1	0.032	t-test	*
			Vimentin	<0.001		***
			ZEB1	<0.001		***
			TWIST	0.015		*
			SLUG	0.011		*
			SNAIL	<0.001		***
		HCC4006	CDH1	<0.001	t-test	***
			Vimentin	0.002		**
			ZEB1	0.004		**
			TWIST	0.074		
			SLUG	0.049		*
			SNAIL	0.094		
		HCC366	CDH1	0.012	t-test	**
			Vimentin	<0.001		***
			ZEB1	<0.001		***
			TWIST	0.051		
			SLUG	0.01		**
			SNAIL	0.035		*
**Fig 2B**	**Cleaved PARP**	H1975	Volasertib_vs_Control	1	ANOVA	
			TGF.Volasertib_vs_TGF.Control	0.000796		***
			TGF.Volasertib_vs_noTGF.Volasertib	0.00056		***
			interaction	0.021599		*
		HCC366	Volasertib_vs_Control	0.00576	ANOVA	**
			TGF.Volasertib_vs_TGF.Control	0.0395		*
			TGF.Volasertib_vs_noTGF.Volasertib	0.21257		

			interaction	1		
		HCC4006	Volasertib_vs_Control	3.77E-05	ANOVA	***
			TGF.Volasertib_vs_TGF.Control	1.30E-06		***
			TGF.Volasertib_vs_noTGF.Volasertib	0.00236		**
			interaction	0.01889		*
	**yH2AX**	H1975	Volasertib_vs_Control	1.81E-05	ANOVA	***
			TGF.Volasertib_vs_TGF.Control	4.03E-12		***
			TGF.Volasertib_vs_noTGF.Volasertib	2.20E-12		***
			interaction	2.20E-10		***
		HCC366	Volasertib_vs_Control	0.150951	ANOVA	
			TGF.Volasertib_vs_TGF.Control	0.000475		***
			TGF.Volasertib_vs_noTGF.Volasertib	0.000594		***
			interaction	0.054048		.
		HCC4006	Volasertib_vs_Control	0.77923	ANOVA	
			TGF.Volasertib_vs_TGF.Control	0.00434		**
			TGF.Volasertib_vs_noTGF.Volasertib	0.04142		*
			interaction	0.14381		
**Fig 2C**	**Apo BrdU**	H1975	Volasertib_vs_Control	0.000512	ANOVA	***
			TGF.Volasertib_vs_TGF.Control	3.68E-08		***
			TGF.Volasertib_vs_noTGF.Volasertib	3.95E-07		***
			interaction	7.47E-06		***
		HCC366	Volasertib_vs_Control	0.00222	ANOVA	**
			TGF.Volasertib_vs_TGF.Control	1.88E-08		***
			TGF.Volasertib_vs_noTGF.Volasertib	7.77E-08		***
			interaction	1.82E-06		***

		HCC4006	Volasertib_vs_Control	0.00121	ANOVA	**
			TGF.Volasertib_vs_TGF.Control	2.58E-08		***
			TGF.Volasertib_vs_noTGF.Volasertib	1.79E-07		***
			interaction	3.32E-06		***
**Fig 2D**	**Comet assay**	H1975	Volasertib_vs_Control	0.002325	ANOVA	**
			TGF.Volasertib_vs_TGF.Control	2.00E-16		***
			TGF.Volasertib_vs_noTGF.Volasertib	1.34E-10		***
			interaction	0.000371		***
		HCC366	Volasertib_vs_Control	0.60656	ANOVA	
			TGF.Volasertib_vs_TGF.Control	4.61E-09		***
			TGF.Volasertib_vs_noTGF.Volasertib	4.41E-05		***
			interaction	0.00385		**
		HCC4006	Volasertib_vs_Control	0.176	ANOVA	
			TGF.Volasertib_vs_TGF.Control	1.33E-06		***
			TGF.Volasertib_vs_noTGF.Volasertib	2.39E-05		***
			interaction	0.102		
**Fig 3C**	**Protein expression**		MET Y1234/35	0.0357	t-test	*
			MET	0.9999		
			FAK Y925	0.3929		
			FAK Y397	0.0357		*
			FAK	0.2857		
			Src Y416	0.5714		
			Src	0.8571		
			AKT S473	0.0714		
			AKT	0.7857		
**Fig 3D**	**Protein expression**		MET Y1234/35	0.09	t-test	
			MET	0.6		

			FAK Y925	0.6		
			FAK Y397	0.8		
			FAK	0.8		
			Src Y416	0.999		
			Src	0.999		
**Fig. 4C**	**Apo BrdU**	HCC366	Volasertib_vs_Control	1	ANOVA	
			Combination_vs_Control	3.19E-06		***
			Combination_vs_Volasertib	6.77E-06		***
			Combination_vs_Tepotinib	3.05E-06		***
			interaction	8.77E-05		***
		H1792	Volasertib_vs_Control	0.013325	ANOVA	*
			Combination_vs_Control	6.16E-06		***
			Combination_vs_Volasertib	0.000128		***
			Combination_vs_Tepotinib	9.44E-06		***
			interaction	0.002559		**
		H1975	Volasertib_vs_Control	0.271048	ANOVA	
			Combination_vs_Control	0.000143		***
			Combination_vs_Volasertib	0.001257		**
			Combination_vs_Tepotinib	0.000139		***
			interaction	0.010998		*
		Calu6	Volasertib_vs_Control	8.66E-06	ANOVA	***
			Combination_vs_Control	3.20E-07		***
			Combination_vs_Volasertib	0.000904		***
			Combination_vs_Tepotinib	3.63E-07		***
			interaction	0.011249		*
**Fig. 4D**	**Cleaved Caspase3**	HCC366	Volasertib_vs_Control	1	ANOVA	
			Combination_vs_Control	0.00531		**

			Combination_vs_Volasertib	0.00962		**
			Combination_vs_Tepotinib	0.00814		**
			interaction	0.05463		.
		H1792	Volasertib_vs_Control	0.02811	ANOVA	*
			Combination_vs_Control	0.00203		**
			Combination_vs_Volasertib	0.02753		*
			Combination_vs_Tepotinib	0.0032		**
			interaction	0.19835		
		H1975	Volasertib_vs_Control	0.09584	ANOVA	.
			Combination_vs_Control	0.00675		**
			Combination_vs_Volasertib	0.07058		.
			Combination_vs_Tepotinib	0.00748		**
			interaction	0.24547		
		Calu6	Volasertib_vs_Control	0.2285	ANOVA	
			Combination_vs_Control	0.0195		*
			Combination_vs_Volasertib	0.1763		
			Combination_vs_Tepotinib	0.031		*
			interaction	0.8195		
	**Cleaved PARP**	HCC366	Volasertib_vs_Control	0.1525	ANOVA	
			Combination_vs_Control	0.0456		*
			Combination_vs_Volasertib	1		
			Combination_vs_Tepotinib	0.0537		.
			interaction	1		
		H1792	Volasertib_vs_Control	0.003131	ANOVA	**
			Combination_vs_Control	0.000629		***
			Combination_vs_Volasertib	0.039002		*
			Combination_vs_Tepotinib	0.000867		***

			interaction	0.275679		
		H1975	Volasertib_vs_Control	0.029025	ANOVA	*
			Combination_vs_Control	0.00033		***
			Combination_vs_Volasertib	0.001431		**
			Combination_vs_Tepotinib	0.000283		***
			interaction	0.004407		**
		Calu6	Volasertib_vs_Control	0.000312	ANOVA	***
			Combination_vs_Control	4.33E-05		***
			Combination_vs_Volasertib	0.001763		**
			Combination_vs_Tepotinib	4.53E-05		***
			interaction	0.007484		**
	**y-H2AX**	HCC366	Volasertib_vs_Control	1	ANOVA	
			Combination_vs_Control	0.0459		*
			Combination_vs_Volasertib	0.0423		*
			Combination_vs_Tepotinib	0.0299		*
			interaction	0.092		.
		H1792	Volasertib_vs_Control	0.009253	ANOVA	**
			Combination_vs_Control	0.000445		***
			Combination_vs_Volasertib	0.00483		**
			Combination_vs_Tepotinib	0.000543		***
			interaction	0.025223		*
		H1975	Volasertib_vs_Control	0.0642	ANOVA	.
			Combination_vs_Control	0.00699		**
			Combination_vs_Volasertib	0.11323		
			Combination_vs_Tepotinib	0.00808		**
			interaction	0.39664		

		Calu6	Volasertib_vs_Control	0.002413	ANOVA	**
			Combination_vs_Control	0.000269		***
			Combination_vs_Volasertib	0.007353		**
			Combination_vs_Tepotinib	0.000681		***
			interaction	0.241679		
**Fig. 4E**	**Clonogenic assay**	HCC366	Volasertib_vs_Control	1	ANOVA	
			Combination_vs_Control	0.00817		**
			Combination_vs_Volasertib	0.04413		*
			Combination_vs_Tepotinib	0.02175		*
			interaction	0.45542		
		H1792	Volasertib_vs_Control	6.95E-05	ANOVA	***
			Combination_vs_Control	6.57E-06		***
			Combination_vs_Volasertib	0.0444		*
			Combination_vs_Tepotinib	2.50E-05		***
			interaction	1		
		H1975	Volasertib_vs_Control	0.0149	ANOVA	*
			Combination_vs_Control	0.00078		***
			Combination_vs_Volasertib	0.19207		
			Combination_vs_Tepotinib	0.00104		**
			interaction	0.78894		
		Calu6	Volasertib_vs_Control	8.86E-09	ANOVA	***
			Combination_vs_Control	3.13E-09		***
			Combination_vs_Volasertib	0.0157		*
			Combination_vs_Tepotinib	1.66E-08		***
			interaction	0.7348		
**Fig. 4F**	**Apo BrdU**	HCC366	PLK1_vs_NT	8.86E-06	ANOVA	***
			Combination_vs_NT	1.19E-07		***

			Combination_vs_PLK1	8.42E-05		***
			Combination_vs_MET	1.85E-06		***
			interaction	0.427		
		H1792	PLK1_vs_NT	2.47E-08	ANOVA	***
			Combination_vs_NT	3.07E-10		***
			Combination_vs_PLK1	2.75E-07		***
			Combination_vs_MET	2.30E-09		***
			interaction	0.000997		***
		H1975	PLK1_vs_NT	1.50E-08	ANOVA	***
			Combination_vs_NT	3.37E-10		***
			Combination_vs_PLK1	7.20E-07		***
			Combination_vs_MET	1.94E-09		***
			interaction	0.00207		**
		Calu6	PLK1_vs_NT	1.93E-07	ANOVA	***
			Combination_vs_NT	5.25E-10		***
			Combination_vs_PLK1	8.69E-08		***
			Combination_vs_MET	7.11E-10		***
			interaction	2.33E-06		***
**Fig. 4G**	**Cleaved Caspase3**	HCC366	PLK1_vs_NT	0.73401	ANOVA	
			Combination_vs_NT	0.00938		**
			Combination_vs_PLK1	0.00406		**
			Combination_vs_MET	0.00694		**
			interaction	0.01184		*
		H1792	PLK1_vs_NT	0.000232	ANOVA	***
			Combination_vs_NT	0.000123		***
			Combination_vs_PLK1	0.155194		

			Combination_vs_MET	0.000132		***
			interaction	0.56097		
		H1975	PLK1_vs_NT	0.004391	ANOVA	**
			Combination_vs_NT	8.76E-05		***
			Combination_vs_PLK1	0.000544		***
			Combination_vs_MET	8.80E-05		***
			interaction	0.002129		**
		Calu6	PLK1_vs_NT	0.013048	ANOVA	*
			Combination_vs_NT	0.000561		***
			Combination_vs_PLK1	0.005456		**
			Combination_vs_MET	0.000479		***
			interaction	0.015396		*
	**Cleaved PARP**	HCC366	PLK1_vs_NT	0.22349	ANOVA	
			Combination_vs_NT	0.00191		**
			Combination_vs_PLK1	0.00613		**
			Combination_vs_MET	0.00231		**
			interaction	0.02848		*
		H1792	PLK1_vs_NT	0.0354	ANOVA	*
			Combination_vs_NT	0.0176		*
			Combination_vs_PLK1	1		
			Combination_vs_MET	0.0189		*
			interaction	1		
		H1975	PLK1_vs_NT	0.947	ANOVA	
			Combination_vs_NT	0.111		
			Combination_vs_PLK1	0.552		
			Combination_vs_MET	0.119		
			interaction	1		

		Calu6	PLK1_vs_NT	0.0289	ANOVA	*
			Combination_vs_NT	0.013		*
			Combination_vs_PLK1	1		
			Combination_vs_MET	0.0102		*
			interaction	1		
	**y-H2AX**	HCC366	PLK1_vs_NT	2.52E-07	ANOVA	***
			Combination_vs_NT	1.31E-08		***
			Combination_vs_PLK1	1.77E-07		***
			Combination_vs_MET	1.78E-08		***
			interaction	1.30E-06		***
		H1792	PLK1_vs_NT	1.25E-06	ANOVA	***
			Combination_vs_NT	2.56E-07		***
			Combination_vs_PLK1	2.22E-05		***
			Combination_vs_MET	4.26E-07		***
			interaction	0.000535		***
		H1975	PLK1_vs_NT	7.61E-07	ANOVA	***
			Combination_vs_NT	8.18E-08		***
			Combination_vs_PLK1	2.45E-06		***
			Combination_vs_MET	1.30E-07		***
			interaction	3.18E-05		***
		Calu6	PLK1_vs_NT	0.001325	ANOVA	**
			Combination_vs_NT	0.000115		***
			Combination_vs_PLK1	0.002431		**
			Combination_vs_MET	0.000172		***
			interaction	0.021877		*
**Fig. 5A**	**Tumor growth curve**	TC402	Volasertib vs Control	0.0392	Tukey method	*

			Tepotinib vs Control	0.2886		
			Combination vs Control	<0.001		***
			Tepotinib vs Volserib	0.8428		
			Combination vs Volasertib	0.0187		*
			Combination vs Tepotinib	0.0014		**
		TC424	Volasertib vs Control	<0.001	Tukey method	***
			Tepotinib vs Control	0.0257		*
			Combination vs Control	<0.001		***
			Tepotinib vs Volserib	0.157		
			Combination vs Volasertib	0.9827		
			Combination vs Tepotinib	0.0766		.
**Fig. 5B**	**Change in tumor volume**		TC424.Volasertib_vs_Control	1.91E-07	ANOVA	***
			TC424.Combination_vs_Tepotinib	0.00506		**
			TC424.Combination_vs_Volasertib	0.608475		
			TC424.Combination_vs_Control	6.81E-08		***
			TC424.interaction	0.057251		.
			TC402.Volasertib_vs_Control	0.000739		***
			TC402.Combination_vs_Tepotinib	8.87E-06		***
			TC402.Combination_vs_Volasertib	0.000739		***
			TC402.Combination_vs_Control	1.94E-09		***
			TC402.interaction	0.349263		
			TC402.Volasertib_vs_TC424.Volasertib	0.00234		**
**Fig 5C**	**Tumor growth curve**	Calu6	Volasertib vs Control	<0.001	Tukey method	***
			Tepotinib vs Control	0.73798		
			Combination vs Control	<0.001		***
			Tepotinib vs Volserib	0.00582		**
			Combination vs Volasertib	0.99162		

			Combination vs Tepotinib	0.00215		**
		H1975	Volasertib vs Control	0.02012	Tukey method	*
			Tepotinib vs Control	0.97724		
			Combination vs Control	<0.001		***
			Tepotinib vs Volserib	0.00717		**
			Combination vs Volasertib	0.03398		*
			Combination vs Tepotinib	<0.001		***
**Fig 5D**	**Change in tumor volume**		calu6.Volasertib_vs_Control	3.99E-07	ANOVA	***
			calu6.Combination_vs_Tepotinib	2.29E-05		***
			calu6.Combination_vs_Volasertib	0.7611		
			calu6.Combination_vs_Control	1.52E-07		***
			calu6.interaction	0.4665		
			H1975.Volasertib_vs_Control	7.09E-06		***
			H1975.Combination_vs_Tepotinib	4.64E-08		***
			H1975.Combination_vs_Volasertib	0.0283		*
			H1975.Combination_vs_Control	1.63E-09		***
			H1975.interaction	0.3696		
			H1975.Volasertib_vs_calu6.Volasertib	0.3696		
**Fig 5E**	**Tumor growth curve**	TC424	Volasertib vs Control	0.1172	Tukey method	
			Tepotinib vs Control	0.6913		
			Combination vs Control	0.0829		.
			Tepotinib vs Volserib	0.6757		
			Combination vs Volasertib	0.9988		
			Combination vs Tepotinib	0.5819		
		Calu 6	Volasertib vs Control	0.06059	Tukey method	.

			Tepotinib vs Control	0.92375		
			Combination vs Control	0.00548		**
			Tepotinib vs Volserib	0.2416		
			Combination vs Volasertib	0.84643		
			Combination vs Tepotinib	0.0385		*
**Fig. 5G**	**TUNEL Assay**	TC 424	Volasertib_vs_Control	0.015261	ANOVA	*
			Combination_vs_Control	0.000215		***
			Combination_vs_Volasertib	0.192027		
			Combination_vs_Tepotinib	0.001095		**
			interaction	1		
		TC402	Volasertib_vs_Control	0.241	ANOVA	
			Combination_vs_Control	3.07E-10		***
			Combination_vs_Volasertib	2.73E-09		***
			Combination_vs_Tepotinib	2.76E-10		***
			interaction	2.00E-06		***
		Calu 6	Volasertib_vs_Control	0.58992	ANOVA	
			Combination_vs_Control	0.00904		**
			Combination_vs_Volasertib	0.25501		
			Combination_vs_Tepotinib	0.00729		**
			interaction	0.91681		
		H1975	Volasertib_vs_Control	0.725	ANOVA	
			Combination_vs_Control	3.32E-07		***
			Combination_vs_Volasertib	2.44E-06		***
			Combination_vs_Tepotinib	6.66E-08		***
			interaction	8.31E-05		***
**Fig 6A**	**Apo BrdU**	Calu6	pBABE.Volasertib_vs_Control	4.58E-10	ANOVA	***
			pBABE.Combination_vs_Tepotinib	1.33E-15		***

			pBABE.Combination_vs_Volasertib	5.20E-13		***
			pBABE.Combination_vs_Control	2.00E-16		***
			pBABE.interaction	4.58E-10		***
			TPRMet.Volasertib_vs_Control	2.90E-05		***
			TPRMet.Combination_vs_Tepotinib	7.84E-08		***
			TPRMet.Combination_vs_Volasertib	0.00436		**
			TPRMet.Combination_vs_Control	1.12E-07		***
			TPRMet.interaction	0.01909		*
			TPRMet.Volasertib_vs_pBABE.Volasertib	5.12E-09		***
			TPRMet.Combination_vs_pBABE.Combination	5.33E-15		***
		H157	pBABE.Volasertib_vs_Control	7.58E-10	ANOVA	***
			pBABE.Combination_vs_Tepotinib	1.50E-11		***
			pBABE.Combination_vs_Volasertib	0.000146		***
			pBABE.Combination_vs_Control	1.50E-11		***
			pBABE.interaction	0.004886		**
			TPRMet.Volasertib_vs_Control	5.05E-05		***
			TPRMet.Combination_vs_Tepotinib	2.68E-07		***
			TPRMet.Combination_vs_Volasertib	0.004082		**
			TPRMet.Combination_vs_Control	2.33E-07		***
			TPRMet.interaction	0.03687		*
			TPRMet.Volasertib_vs_pBABE.Volasertib	1.19E-06		***
			TPRMet.Combination_vs_pBABE.Combination	1.64E-07		***
		H1355	pBABE.Volasertib_vs_Control	2.84E-07	ANOVA	***
			pBABE.Combination_vs_Tepotinib	4.84E-11		***
			pBABE.Combination_vs_Volasertib	4.77E-05		***
			pBABE.Combination_vs_Control	8.26E-10		***
			pBABE.interaction	8.04E-06		***
			TPRMet.Volasertib_vs_Control	0.0787		.
			TPRMet.Combination_vs_Tepotinib	2.45E-08		***
			TPRMet.Combination_vs_Volasertib	6.02E-07		***

			TPRMet.Combination_vs_Control	6.58E-08		***
			TPRMet.interaction	8.21E-06		***
			TPRMet.Volasertib_vs_pBABE.Volasertib	1.29E-07		***
			TPRMet.Combination_vs_pBABE.Combination	6.49E-06		***
**Fig 6C**	**Clonogenic assay**	Calu 6	pBABE.Volasertib_vs_Control	6.26E-11	ANOVA	***
			pBABE.Combination_vs_Tepotinib	6.34E-11		***
			pBABE.Combination_vs_Volasertib	0.06153		.
			pBABE.Combination_vs_Control	2.20E-11		***
			pBABE.interaction	0.74621		
			TPRMet.Volasertib_vs_Control	8.65E-08		***
			TPRMet.Combination_vs_Tepotinib	1.06E-09		***
			TPRMet.Combination_vs_Volasertib	0.00143		**
			TPRMet.Combination_vs_Control	9.26E-10		***
			TPRMet.interaction	0.02406		*
			TPRMet.Volasertib_vs_pBABE.Volasertib	2.42E-06		***
			TPRMet.Combination_vs_pBABE.Combination	7.10E-05		***
**Fig 7C**	**RTPCR**		CDH1	<0.001	t test	***
			Vimentin	<0.001		***
			ZEB1	<0.001		***
			TWIST	<0.001		***
			SLUG	<0.001		***
			SNAIL	<0.001		***
**Fig 7D**	**Apo BrdU**		Calu6.Volasertib25_vs_Control	1.90E-10	ANOVA	***
			Calu6.Combination_vs_Tepotinib	2.02E-11		***
			Calu6.Combination_vs_Volasertib25	0.0125		*
			Calu6.Combination_vs_Control	2.02E-11		***
			Calu6.interaction	0.0597		.
			Calu6VAR.Volasertib250_vs_Control	7.33E-05		***
			Calu6VAR.Combination_vs_Tepotinib	6.00E-07		***

			Calu6VAR.Combination_vs_Volasertib250	0.0154		*
			Calu6VAR.Combination_vs_Control	6.00E-07		***
			Calu6VAR.interaction	0.0597		.
			Calu6VAR.Volasertib250_vs_Calu6.Volasertib25	9.04E-08		***
**Fig 7F**	**Clonogenic assay**		Calu6.Volasertib25_vs_Control	1.31E-11	ANOVA	***
			Calu6.Combination_vs_Tepotinib	1.31E-11		***
			Calu6.Combination_vs_Volasertib25	0.178014		
			Calu6.Combination_vs_Control	3.98E-12		***
			Calu6.interaction	0.924357		
			Calu6VAR.Volasertib25_vs_Control	0.767331		
			Calu6VAR.Combination25_vs_Tepotinib	0.000114		***
			Calu6VAR.Combination25_vs_Volasertib25	0.000998		***
			Calu6VAR.Combination25_vs_Control	0.000438		***
			Calu6VAR.interaction25	0.005663		**
			Calu6VAR2.Volasertib250_vs_Control	0.17251		
			Calu6VAR2.Combination250_vs_Tepotinib	1.80E-09		***
			Calu6VAR2.Combination250_vs_Volasertib250	2.06E-08		***
			Calu6VAR2.Combination250_vs_Control	1.29E-09		***
			Calu6VAR2.interaction250	9.21E-06		***
			Calu6VAR.Volasertib25_vs_Calu6.Volasertib25	1.78E-11		***

ANOVA, Analysis of variance
