# Supplementary material for: Non‐canonical cMet regulation by vimentin mediates Plk1 inhibitor–induced apoptosis
Source: EMBO Mol Med. 2019 Apr 30;11(5):e9960. doi: 10.15252/emmm.201809960 (PMC6505578; doi:10.15252/emmm.201809960)
Supplement: Supplementary file 2 — Expanded View Figures PDF [file EMMM-11-e9960-s002.pdf]

Expanded View Figures

Figure EV1. Basal expression of proteins that correlate with sensitivity to Plk1 inhibitors.

A–D Sensitivity data for the Plk1 inhibitors BI2536, GSK461364, BRD-K70511574, and GW-843682X were obtained from the Cancer Therapeutics Response Portal v2 database. Protein expression data (reverse phase protein array) were obtained from the MD Anderson Cell Line Project database. Spearman's correlations of area under the curve (AUC) values and protein expression in non–small-cell lung cancer cell lines *in vitro* are shown for those with a Spearman rho coefficient > 0.3 for BI2536 (A), GSK461364 (B), GW-843682X (C), and BRD-K70511574 (D). The color of the bars indicates the *P*-value per the legend in the graph.

E Basal cMet protein expression in non–small-cell lung cancer cell lines compared with AUC values of GW-843682X and BRD-K70511574. The blue line represents linear regression and 95% confidence interval is indicated in dark gray.

© 2019 The Authors

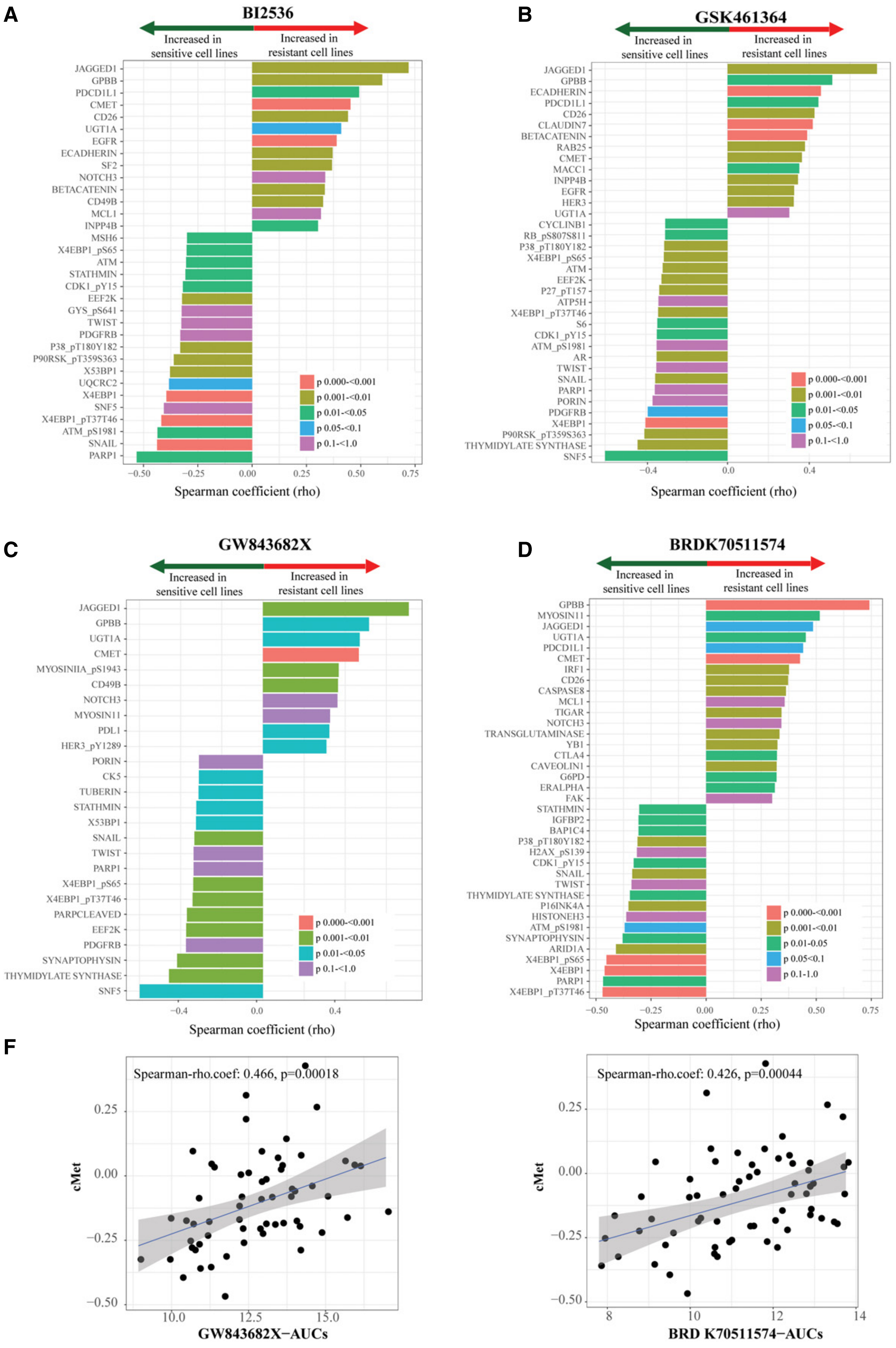

Figure EV1.

 © 2019 The Authors

Figure EV2. cMet, FAK, and Src phosphorylation regulation in epithelial and mesenchymal non–small-cell lung cancer cell lines following Plk1 inhibition.

A Box plots show protein expression of p-cMet (Y1234/35), pFAK (Y397), and pSrc (Y416) measured using reverse phase protein array after treatment with volasertib in non–small-cell lung cancer cell lines. Experiments were performed in triplicate. The median is marked by a horizontal line, the colored boxes are the upper and lower quartiles, and error bars represent standard deviation. FC, fold change.

B Three epithelial (H1975, HCC4006, and HCC366) and two mesenchymal (Calu6 and H1792) NSCLC cell lines were incubated with 50 nM volasertib for 24 h, lysed, and subjected to reverse phase protein array analysis. Experiments were done in triplicate.

C Seventy proteins were differentially regulated between epithelial and mesenchymal NSCLC cell lines after treatment with volasertib, including those involved in the cMet/FAK/Src signaling axis proteins (blue text) and the PI3K/Akt signaling axis (orange text). FDR, false discovery rate.

© 2019 The Authors

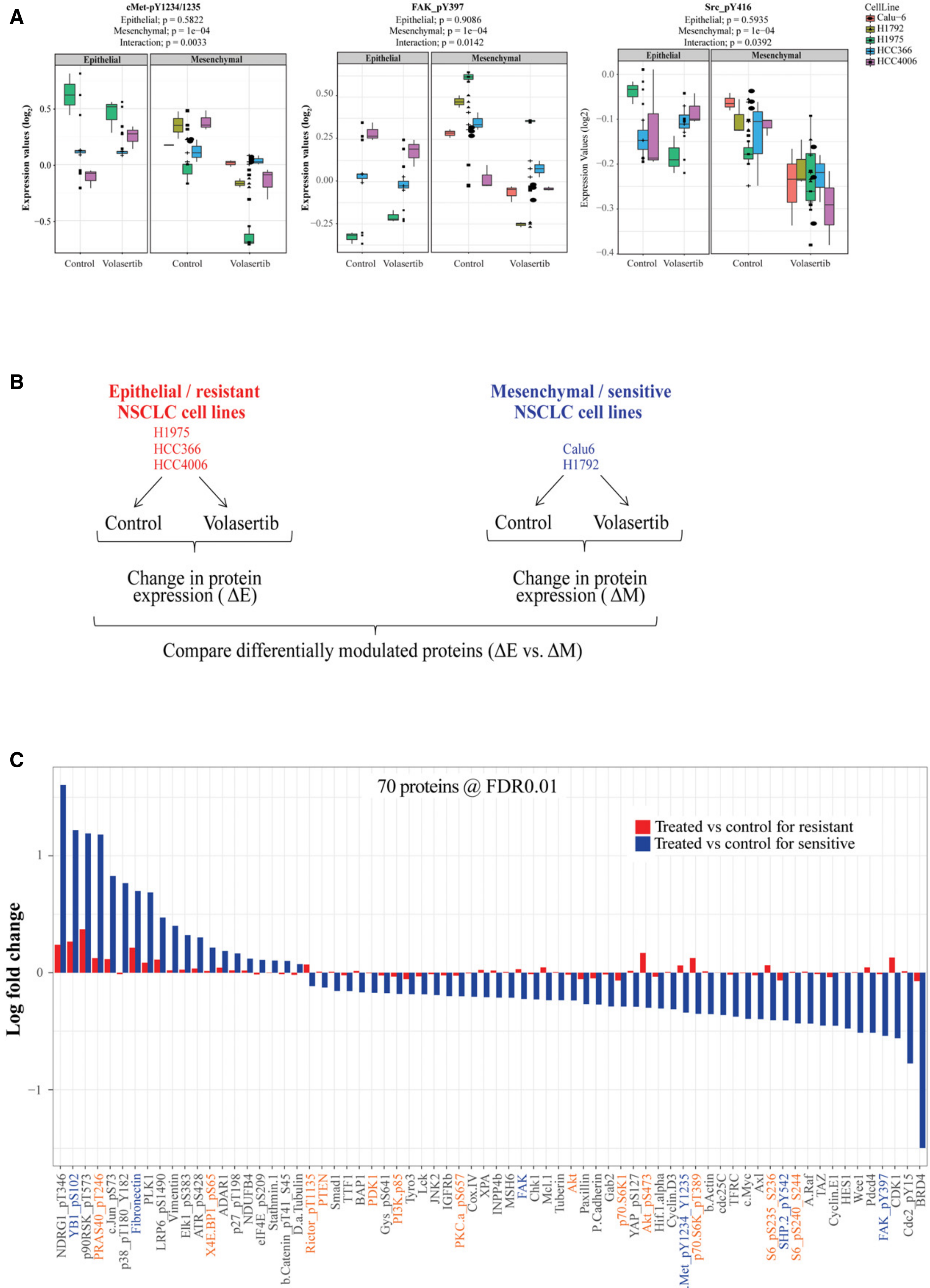

Figure EV2.

 © 2019 The Authors

Figure EV3. Co-targeting of cMet and Plk1 reduces tumor size in non–small-cell lung cancer.

A mRNA was extracted from two untreated patient-derived xenograft (PDX) tumors, and the expression of epithelial-to-mesenchymal transition genes was normalized with respect to GAPDH expression. Data are means ± standard error of the mean from two independent tumors.

B Mice bearing epithelial (red text) and mesenchymal (blue text) non–small-cell lung cancer PDXs were treated with vehicle control, volasertib (30 mg/kg per week intravenously), tepotinib (25 mg/kg per day orally), or the combination for 5 weeks; representative PDX tumors from the end of treatment are pictured.

C Waterfall plots show the percent change in individual PDX tumor volumes at the end of treatment (normalized to day zero), as indicated.

D mRNA was extracted from two untreated xenograft tumors, and the expression of epithelial-to-mesenchymal transition genes was normalized with respect to GAPDH expression. Data are means ± standard error of the mean from two independent tumors.

E Mice bearing epithelial (red text) and mesenchymal (blue text) NSCLC cell line xenografts were treated with vehicle control, volasertib (30 mg/kg per week intravenously), tepotinib (25 mg/kg per day orally), or the combination for 5 weeks; representative xenograft tumors from the end of treatment are pictured at the top.

F Waterfall plots show the percent change in individual xenograft tumor volumes at the end of treatment (normalized to day zero), as indicated.

G Kaplan–Meier survival curves for mice with epithelial H1975 and mesenchymal Calu6 xenografts, with death due to excessive tumor burden as the endpoint. Log-rank (Mantel-Cox) test was used for Kaplan–Meier survival curves.

H Mice bearing non–small-cell lung cancer cell line xenografts (left) were treated for 4 weeks and mice bearing patient-derived xenografts (right) were treated for 5 weeks with vehicle control, volasertib (30 mg/kg per week intravenously), tepotinib (25 mg/kg per day orally), or the combination as indicated. Mouse weight was recorded twice weekly to generate the graphs. Data are mean ± standard error of the mean from 10 mice for each group.

© 2019 The Authors

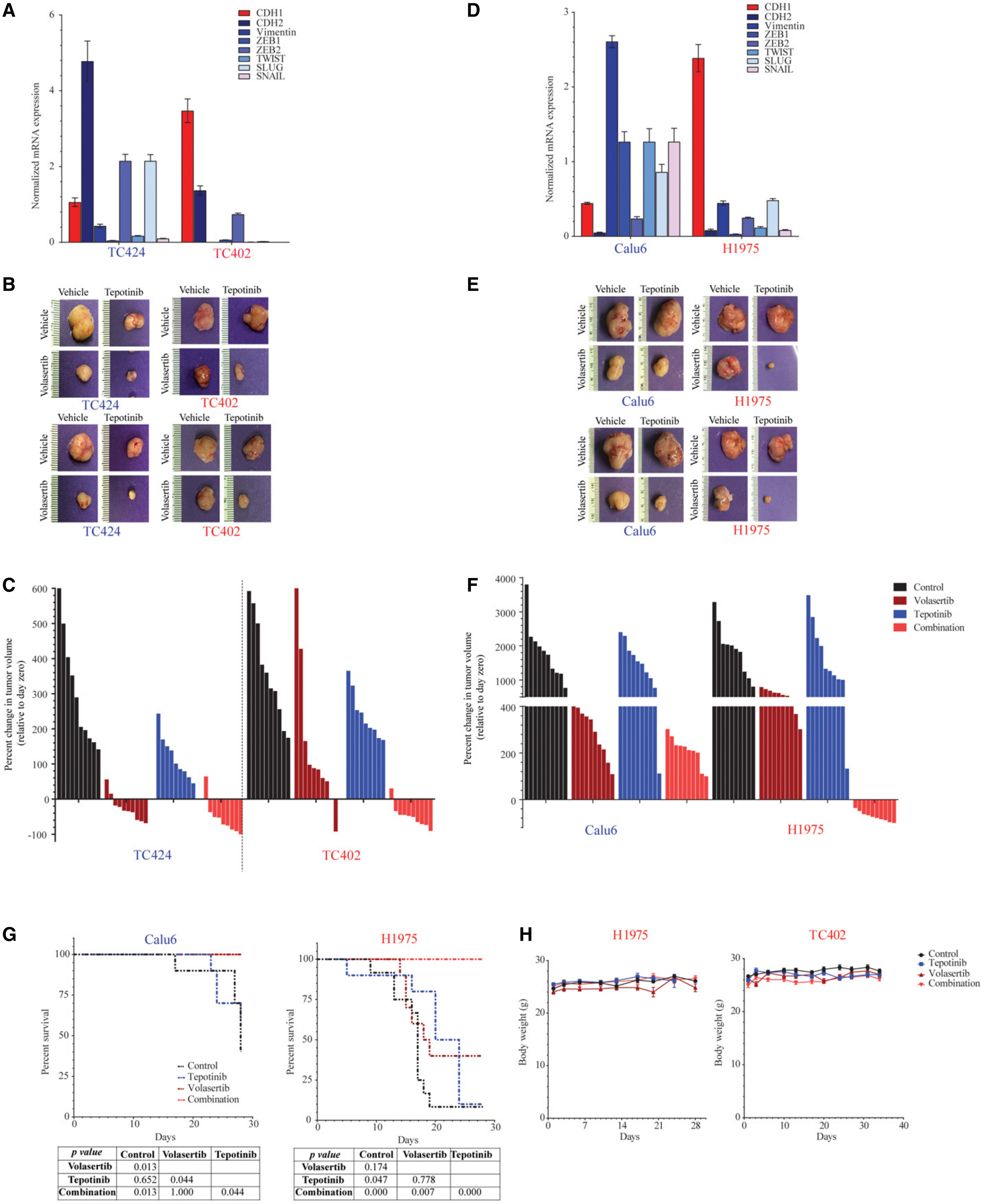

p value	Control	Volasertib	Tepotinib
Volasertib	0.013		
Tepotinib	0.652	0.044	
Combination	0.013	1.000	0.044

p value	Control	Volasertib	Tepotinib
Volasertib	0.174		
Tepotinib	0.047	0.778	
Combination	0.000	0.007	0.000

Figure EV3.

© 2019 The Authors

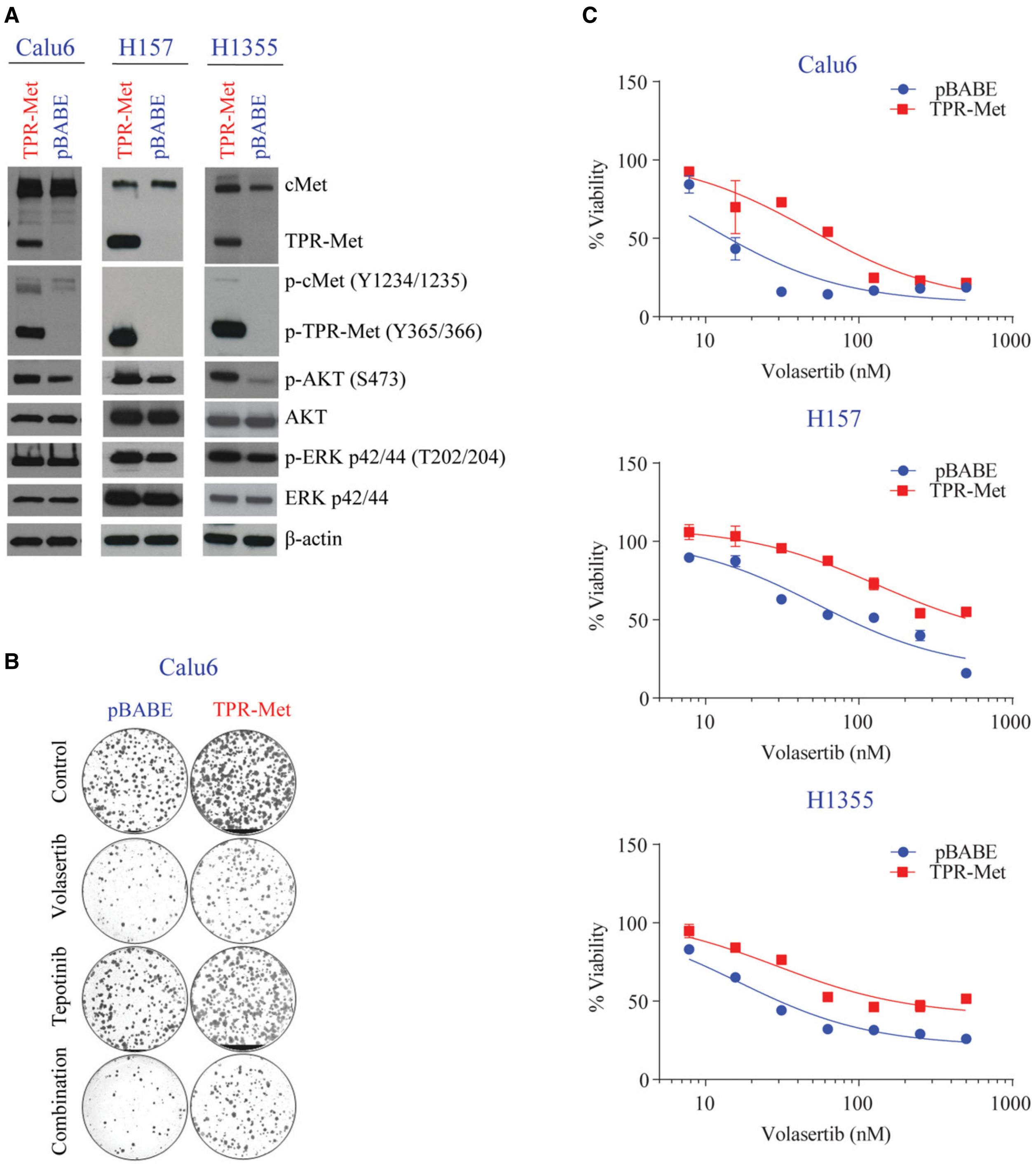

Figure EV4. Expression of the TPR-Met chimeric protein is biologically active and decreases volasertib sensitivity.

A Calu6, H157, and H1355 parental and TPR-Met–expressing cell lines or vector control (pBABE)–expressing cells were harvested, lysed, and subjected to immunoblotting for the indicated proteins. β-Actin was used as a loading control.

B Shown are representative pictures of Calu6 parental and TPR-Met–expressing cell lines treated with 25 nM volasertib, 400 nM tepotinib, both, or vehicle control for 24 h and allowed to grow in drug-free medium for 15–20 days to form colonies, which were then stained with crystal violet and photographed.

C Cell viability of parental and TPR-Met–expressing Calu6, H157, and H1355 cell lines treated with the indicated concentrations of volasertib for 72 h was measured using CellTiter-Glo. Experiments were performed in triplicate, and error bars represent standard deviation.

Source data are available online for this figure.

© 2019 The Authors

Figure EV5. Plk1 regulates vimentin phosphorylation, leading to integrin β1–mediated cMet activation.

A Non–small-cell lung cancer cell lines were treated with 50 nM volasertib in serum-free medium for 24 h. HGF levels were measured in conditioned medium (upper). Data are means ± standard error of the mean from three independent experiments. Non–small-cell lung cancer cell lines were treated as indicated with 25 nM volasertib or 400 nM tepotinib for 24 h. Cells were then harvested, and lysates were immunoblotted for the indicated proteins (lower).

B The indicated mesenchymal cell lines were incubated with 0.5 μg/ml HGF neutralizing antibody or 50 nM volasertib as indicated for 24 h and subjected to immunoblotting.

C NSCLC cell line mRNA expression of vimentin (*VIM*) and integrin β1 (*ITGB1*) and epithelial-to-mesenchymal transition (EMT) scores were obtained from our previous study (22). Spearman's correlations of EMT score and mRNA expression of *VIM* and *ITGB1* in NSCLC cell lines *in vitro*.

D Calu6 and H1792 cell lines were transfected with 10 nM siRNA for 48 h and subsequently treated with 25 nM volasertib for 24 h as indicated. Cells were then harvested, and lysates were immunoblotted for the indicated proteins.

E NSCLC cell lines were transfected with *ITGB1* and Plk1 siRNA for 48 h. Cells were then harvested, and lysates were immunoblotted for the indicated proteins.

F NSCLC cell lines were treated with volasertib in fibronectin-coated (5 μg/cm^2 for 30 min at room temperature) or fibronectin-uncoated plates. Cells were then harvested, and lysates were immunoblotted for the indicated proteins. NT, non-targeting control.

Source data are available online for this figure.

 © 2019 The Authors

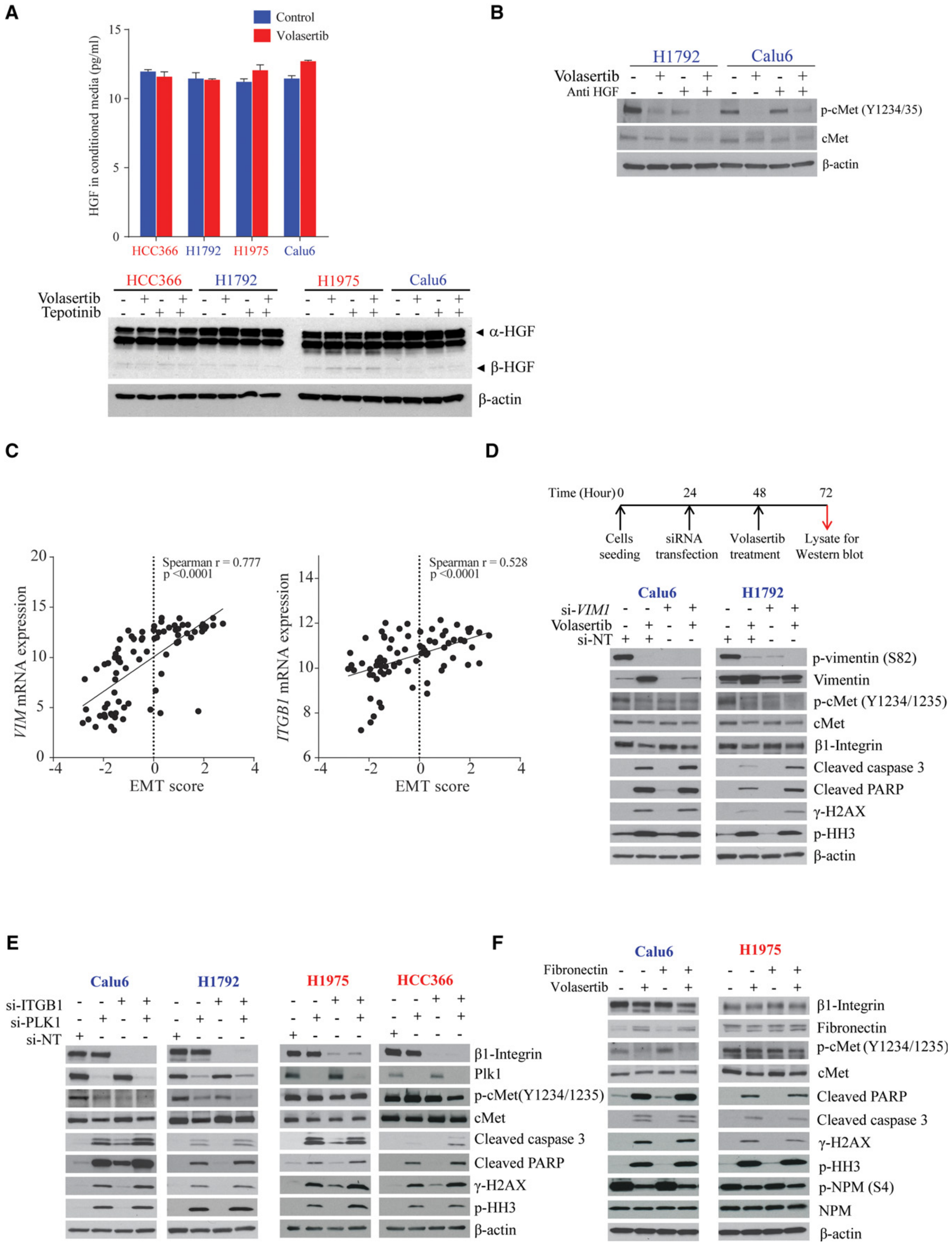
A
Control
Volasertib
HGF in conditioned media (pg/ml)
HCC366
H1792
H1975
Calu6
Volasertib
Tepotinib
α-HGF
β-HGF
β-actin
B
H1792
Calu6
Volasertib
Anti HGF
p-cMet (Y1234/35)
cMet
β-actin
C
Spearman r = 0.777
p <0.0001
VIM mRNA expression
EMT score
Spearman r = 0.528
p <0.0001
ITGB1 mRNA expression
EMT score
D
Time (Hour) 0 24 48 72
Cells seeding
siRNA transfection
Volasertib treatment
Lysate for Western blot
Calu6
H1792
si-VIM1
Volasertib
si-NT
p-vimentin (S82)
Vimentin
p-cMet (Y1234/1235)
cMet
β1-Integrin
Cleaved caspase 3
Cleaved PARP
γ-H2AX
p-HH3
β-actin
E
Calu6
H1792
H1975
HCC366
si-ITGB1
si-PLK1
si-NT
β1-Integrin
Plk1
p-cMet(Y1234/1235)
cMet
Cleaved caspase 3
Cleaved PARP
γ-H2AX
p-HH3
β-actin
F
Calu6
H1975
Fibronectin
Volasertib
β1-Integrin
Fibronectin
p-cMet (Y1234/1235)
cMet
Cleaved PARP
Cleaved caspase 3
γ-H2AX
p-HH3
p-NPM (S4)
NPM
β-actin

Figure EV5.
