# Supplementary material for: Non‐canonical cMet regulation by vimentin mediates Plk1 inhibitor–induced apoptosis
Source: EMBO Mol Med. 2019 Apr 30;11(5):e9960. doi: 10.15252/emmm.201809960 (PMC6505578; doi:10.15252/emmm.201809960)

Source data for Fig. EV5

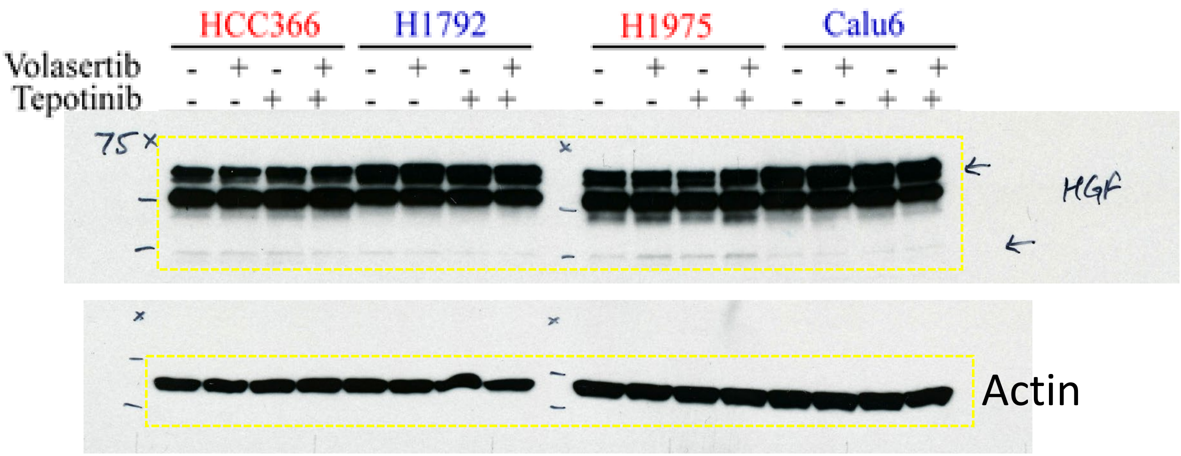

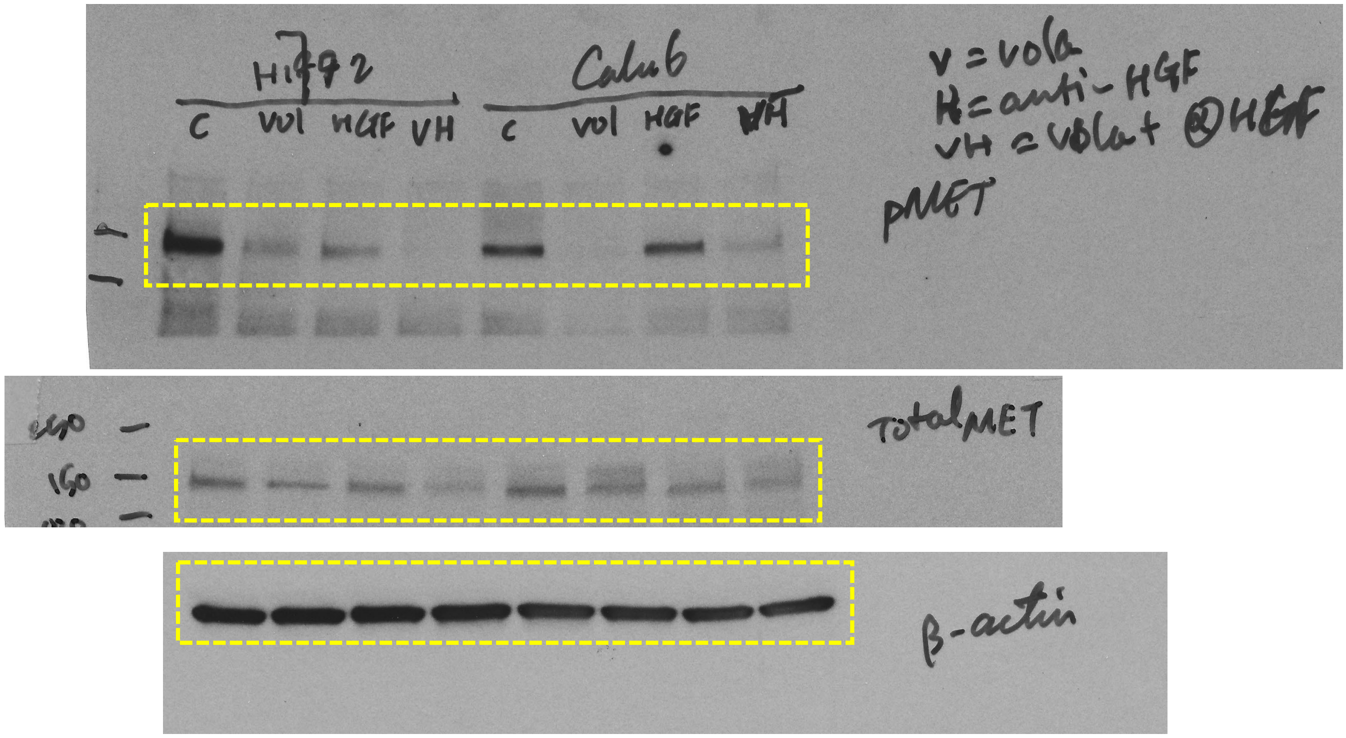

Source data for Fig. EV5 D

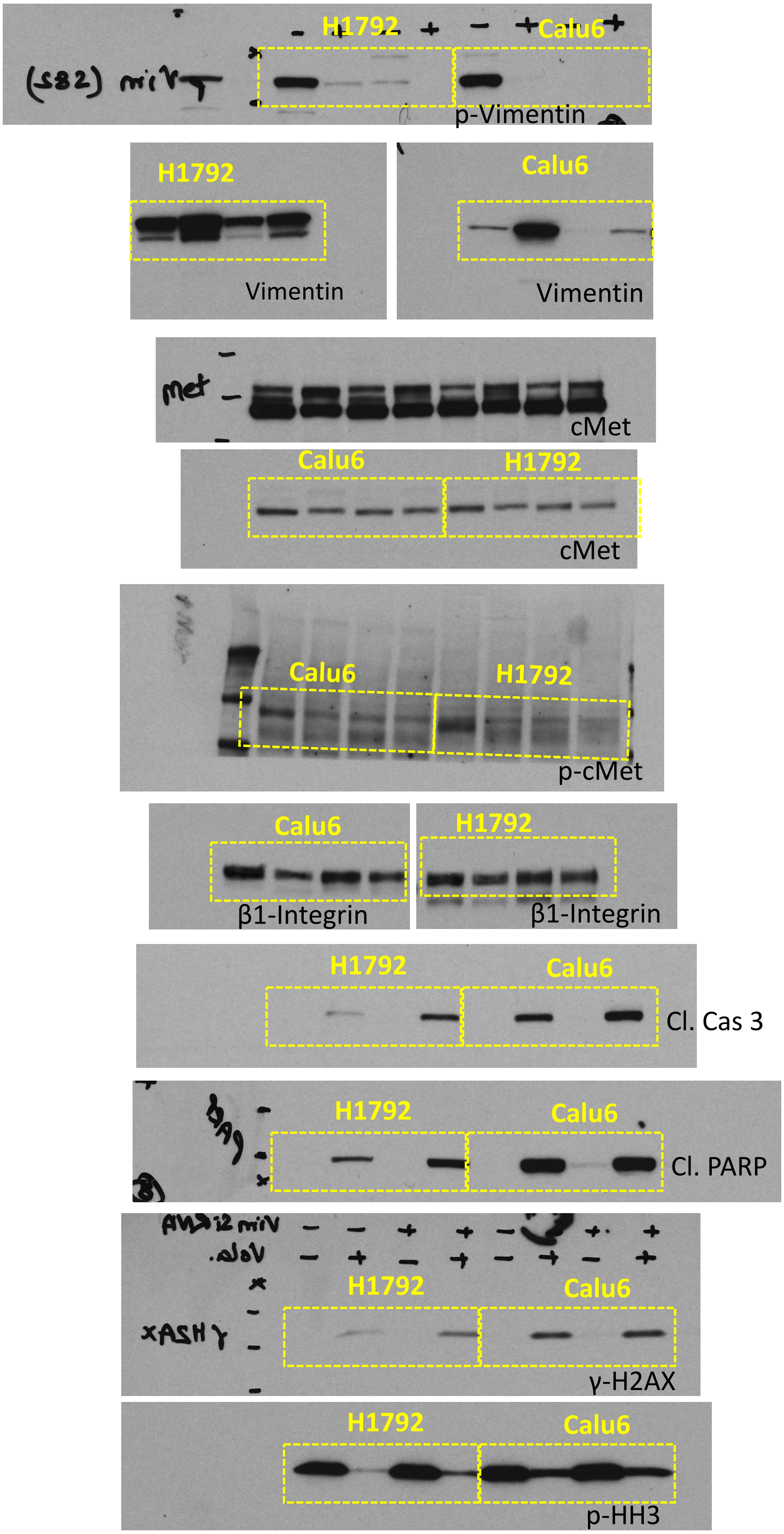

Source data for Fig. EV5 E

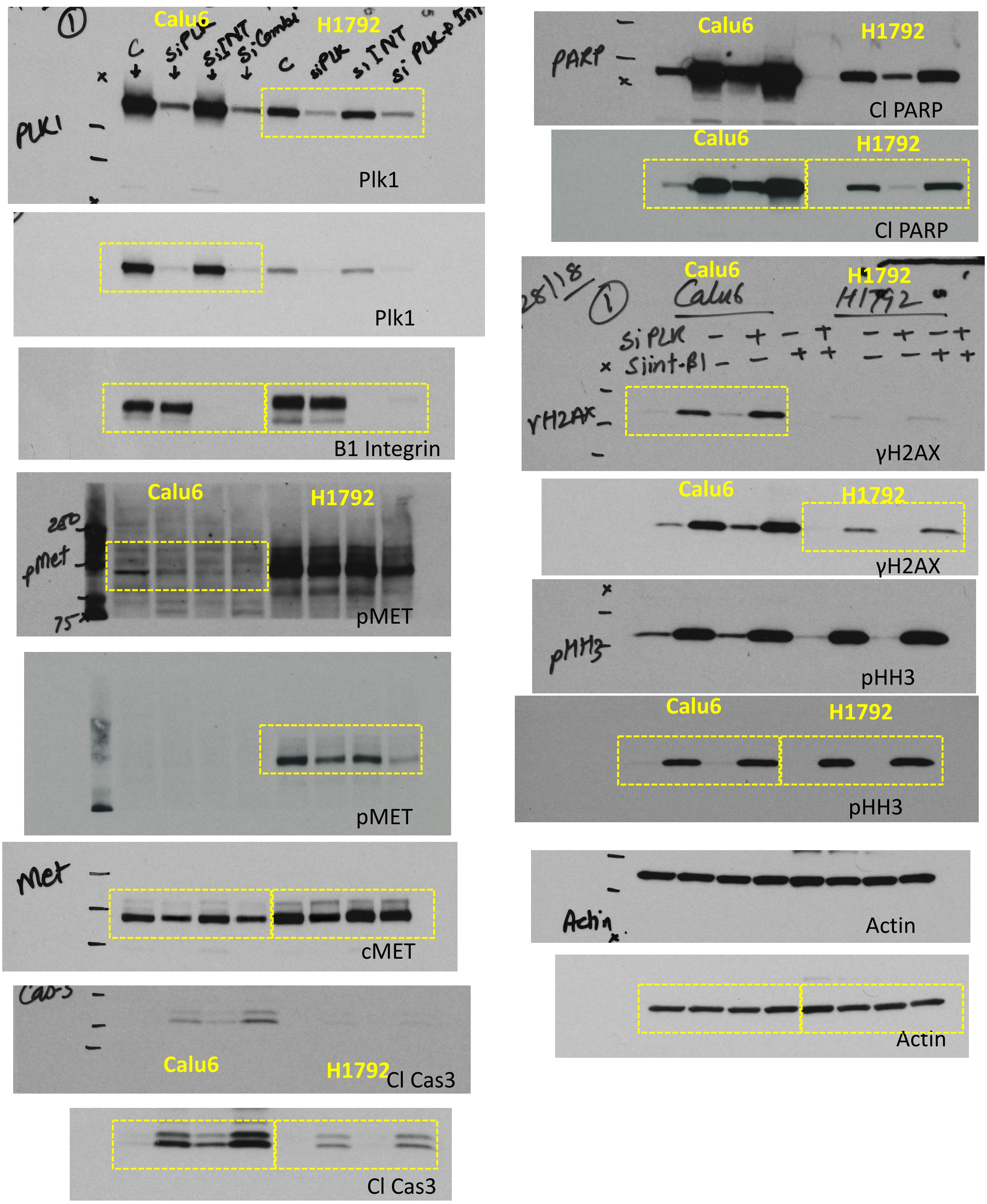

Source data for Fig. EV5 E

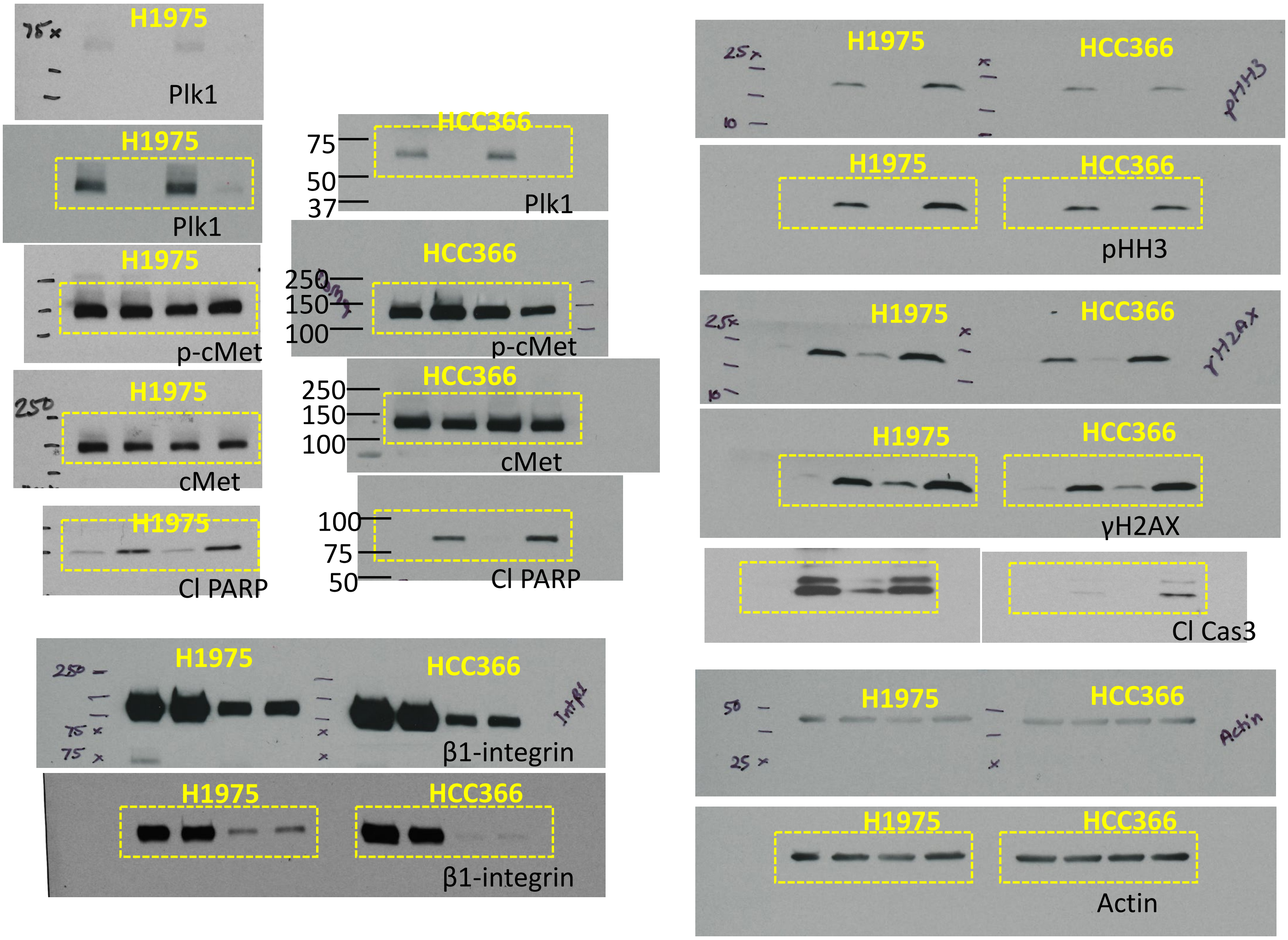

Source data for Fig. EV5 E

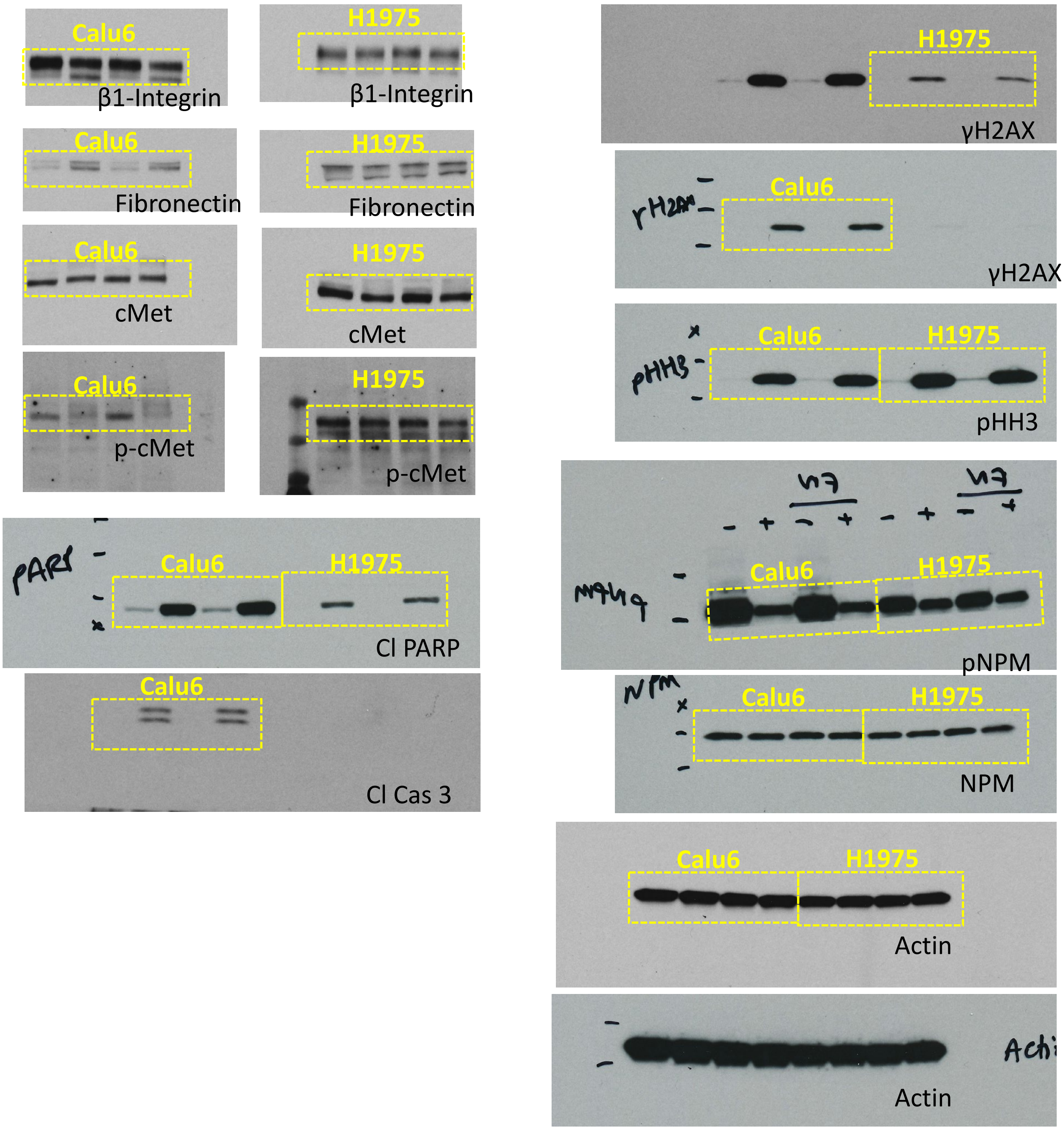

Supplement: Supplementary file 3 — Source Data for Expanded View and Appendix [file EMMM-11-e9960-s009.zip › Source data for EV5 04082019.pdf]
