# Supplementary figures and images for: Non‐canonical cMet regulation by vimentin mediates Plk1 inhibitor–induced apoptosis
Source: EMBO Mol Med. 2019 Apr 30;11(5):e9960. doi: 10.15252/emmm.201809960 (PMC6505578; doi:10.15252/emmm.201809960)

Source data for Appendix Figure 3A

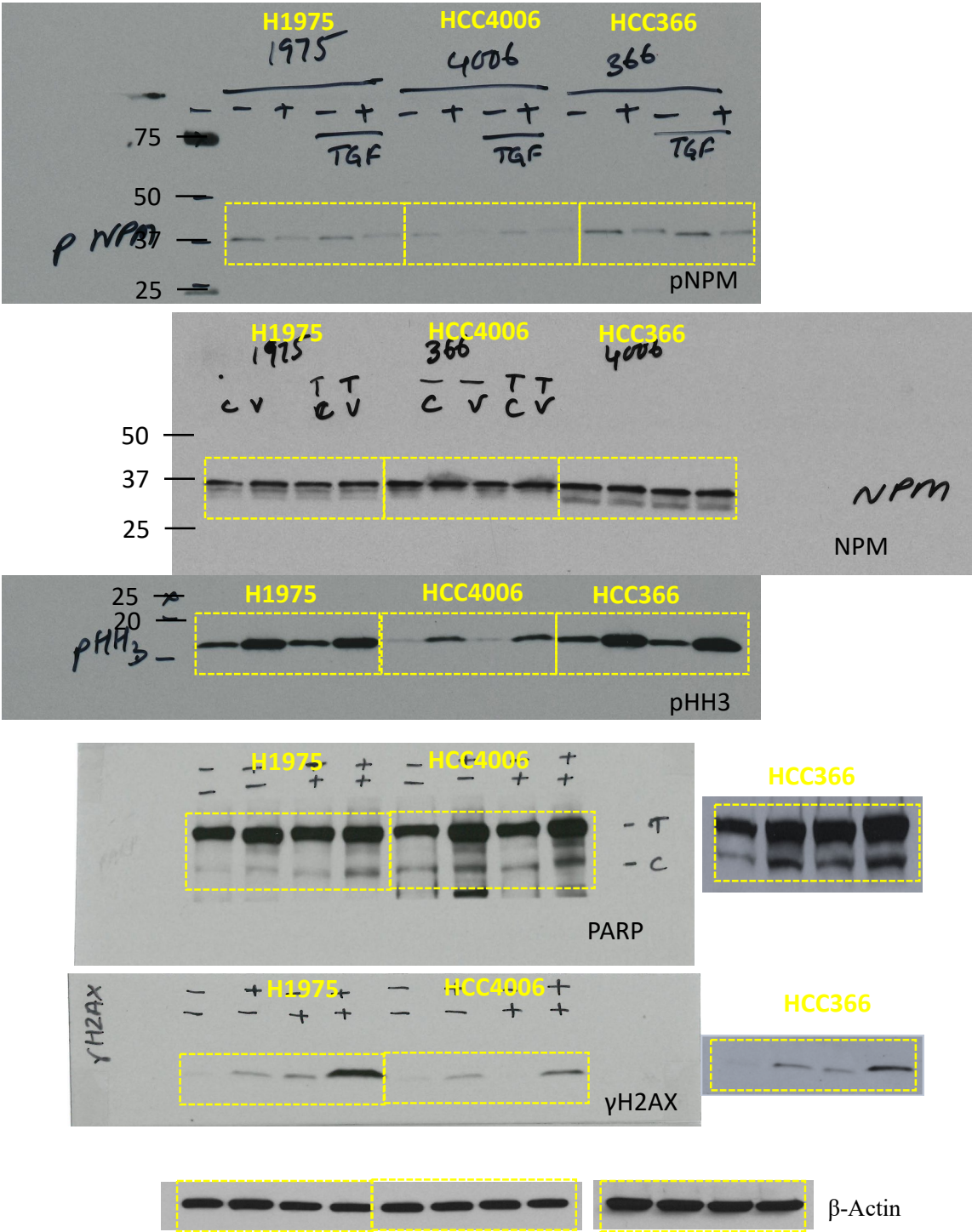

Supplement: Supplementary file 3 — Source Data for Expanded View and Appendix [file EMMM-11-e9960-s009.zip › Appendix Figure 3 Source Data.pdf]

Source data for Fig. EV4

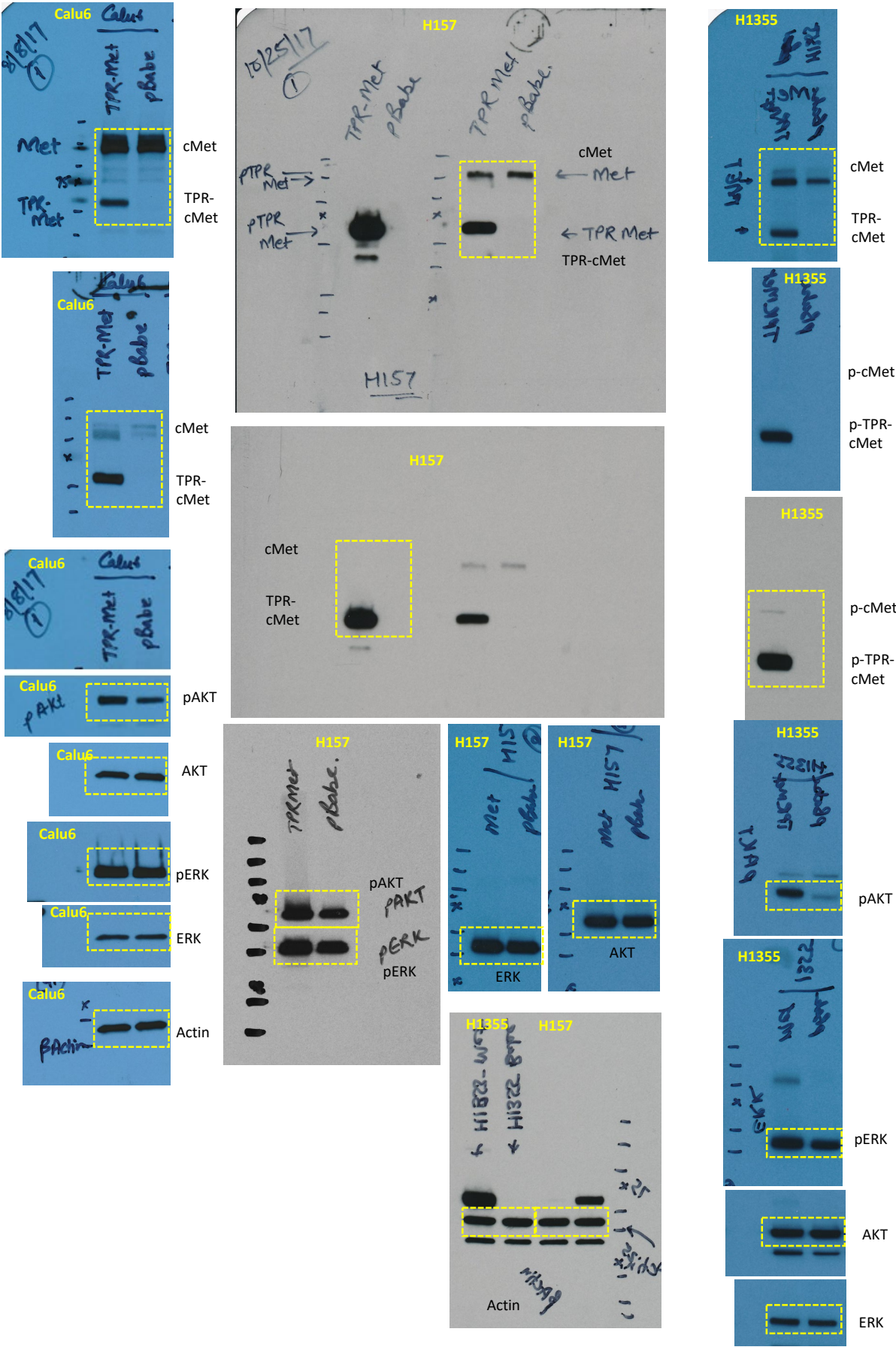

Supplement: Supplementary file 3 — Source Data for Expanded View and Appendix [file EMMM-11-e9960-s009.zip › Source data for EV4.pdf]

Source data for Figure 2

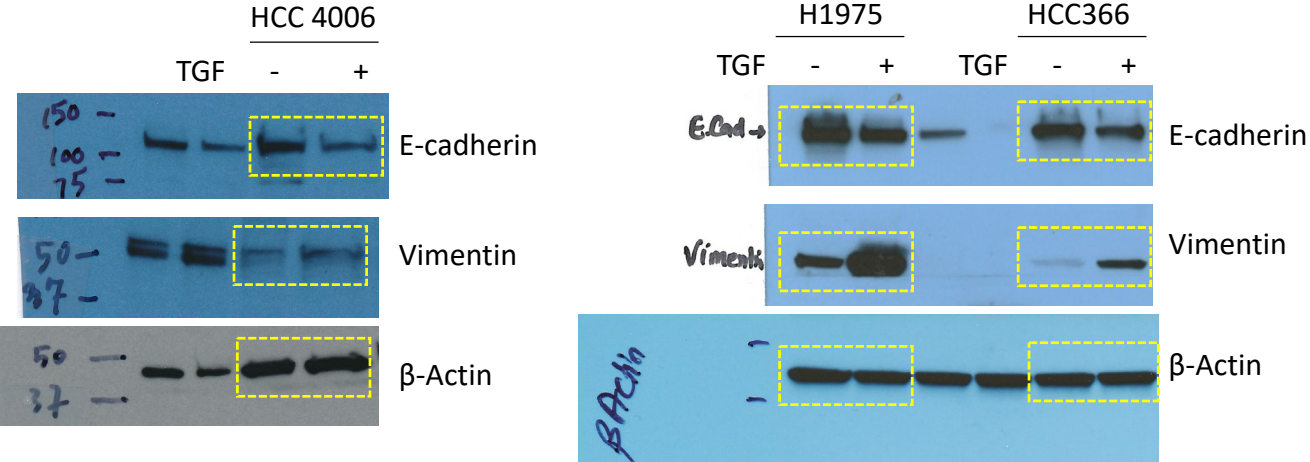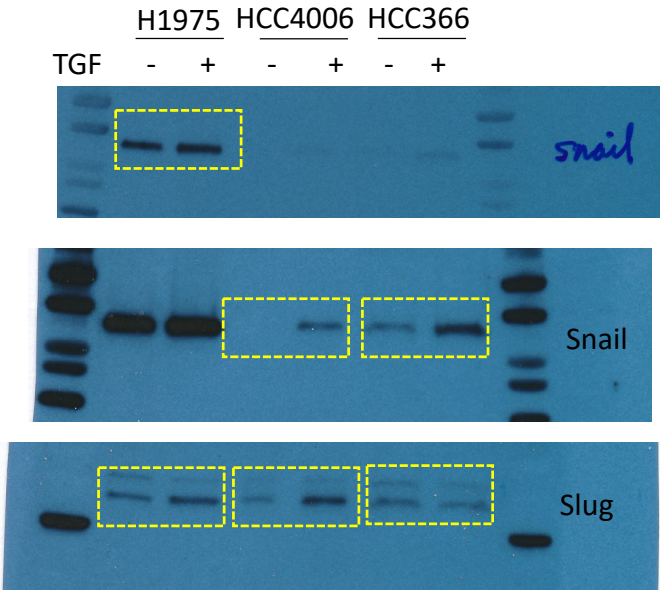

Supplement: Supplementary file 5 — Source Data for Figure 2 [file EMMM-11-e9960-s003.pdf]

Source data for Figure 4D

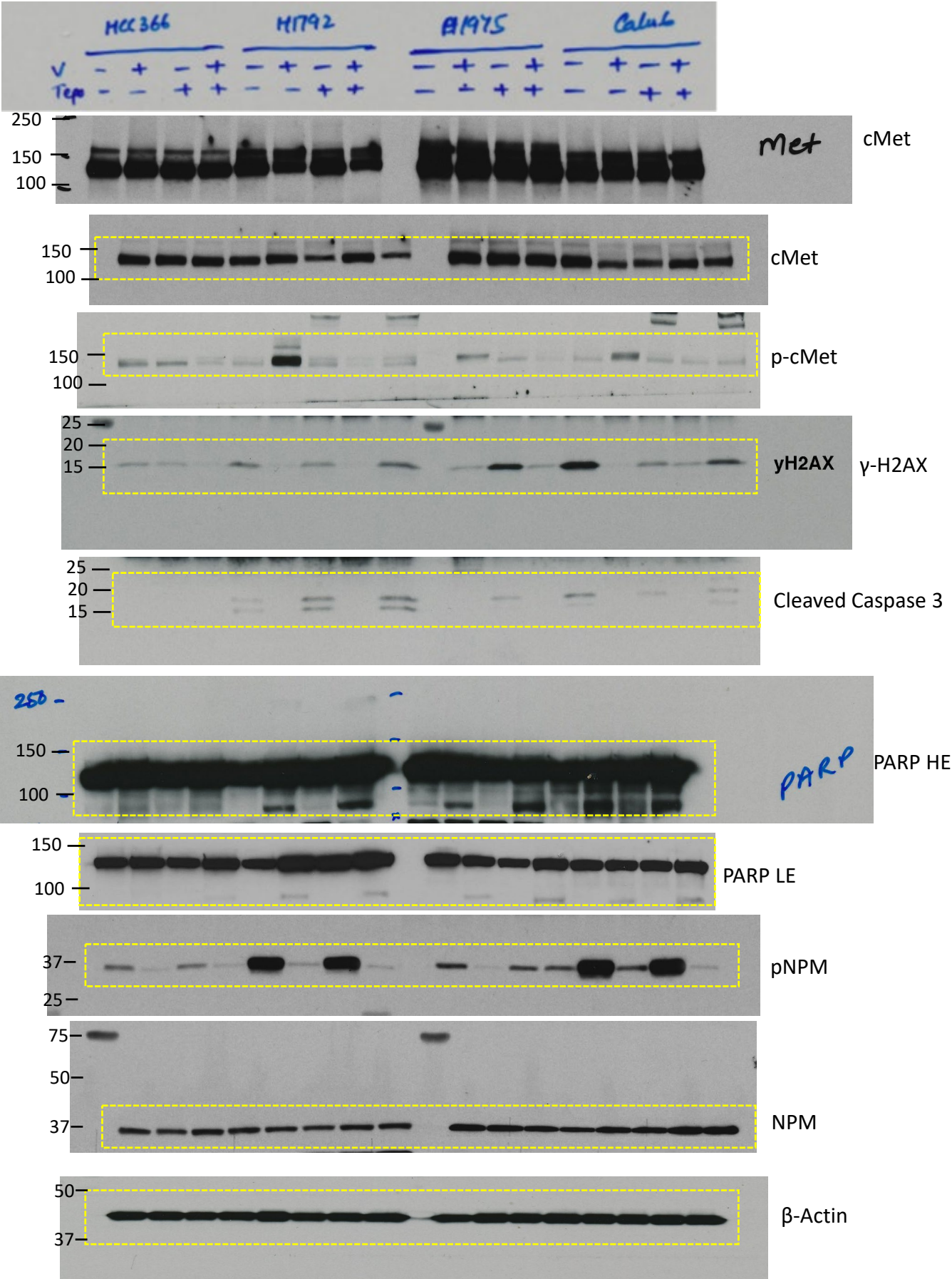

### Source data for Figure 4G

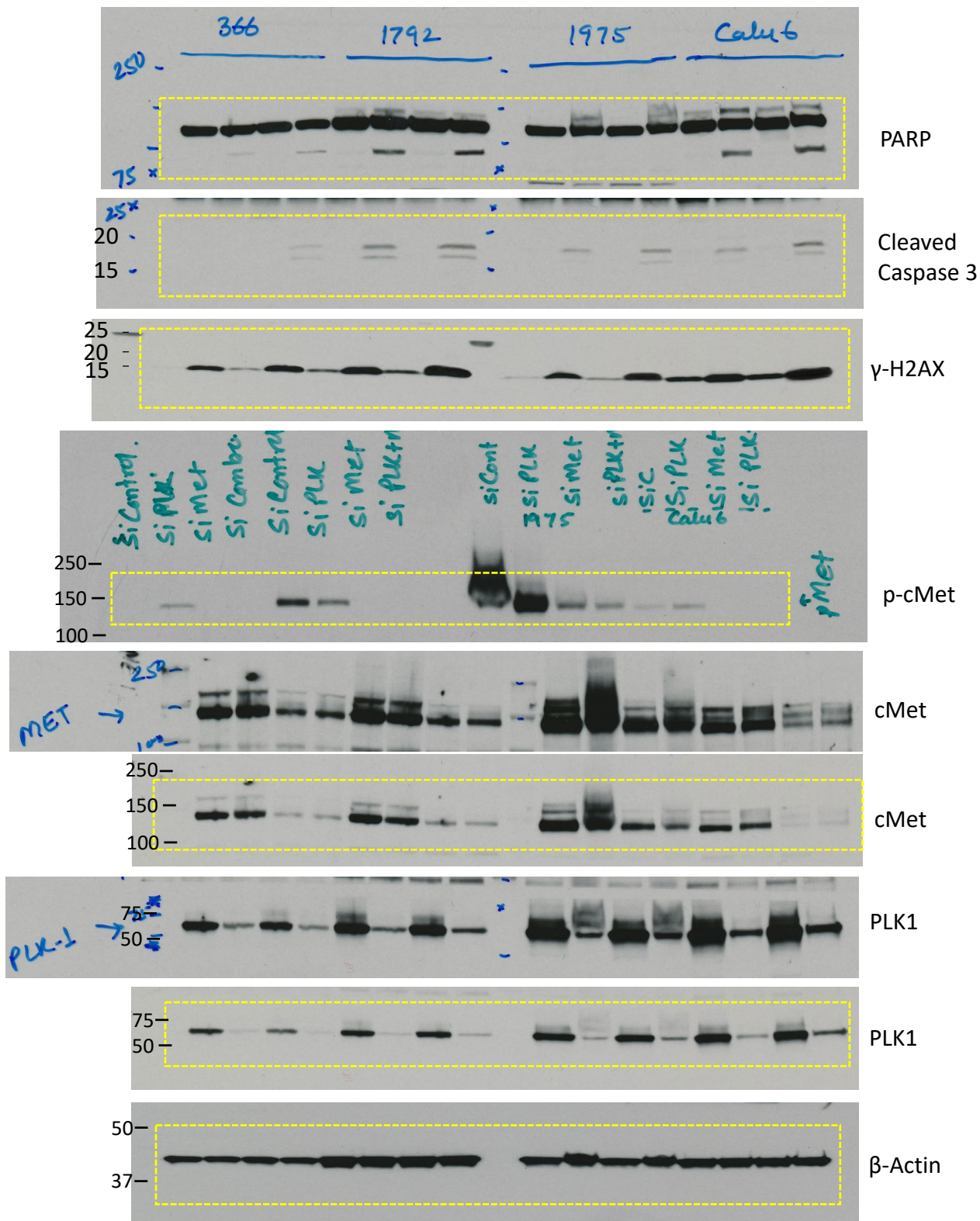

Supplement: Supplementary file 7 — Source Data for Figure 5 [file EMMM-11-e9960-s005.pdf]

Source data for Figure 7C

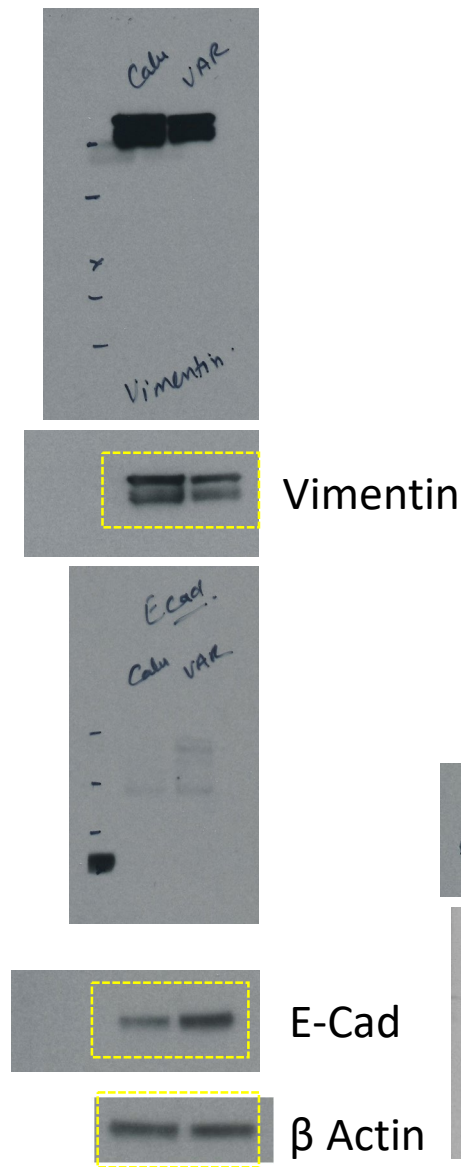

Source data for Figure 7E

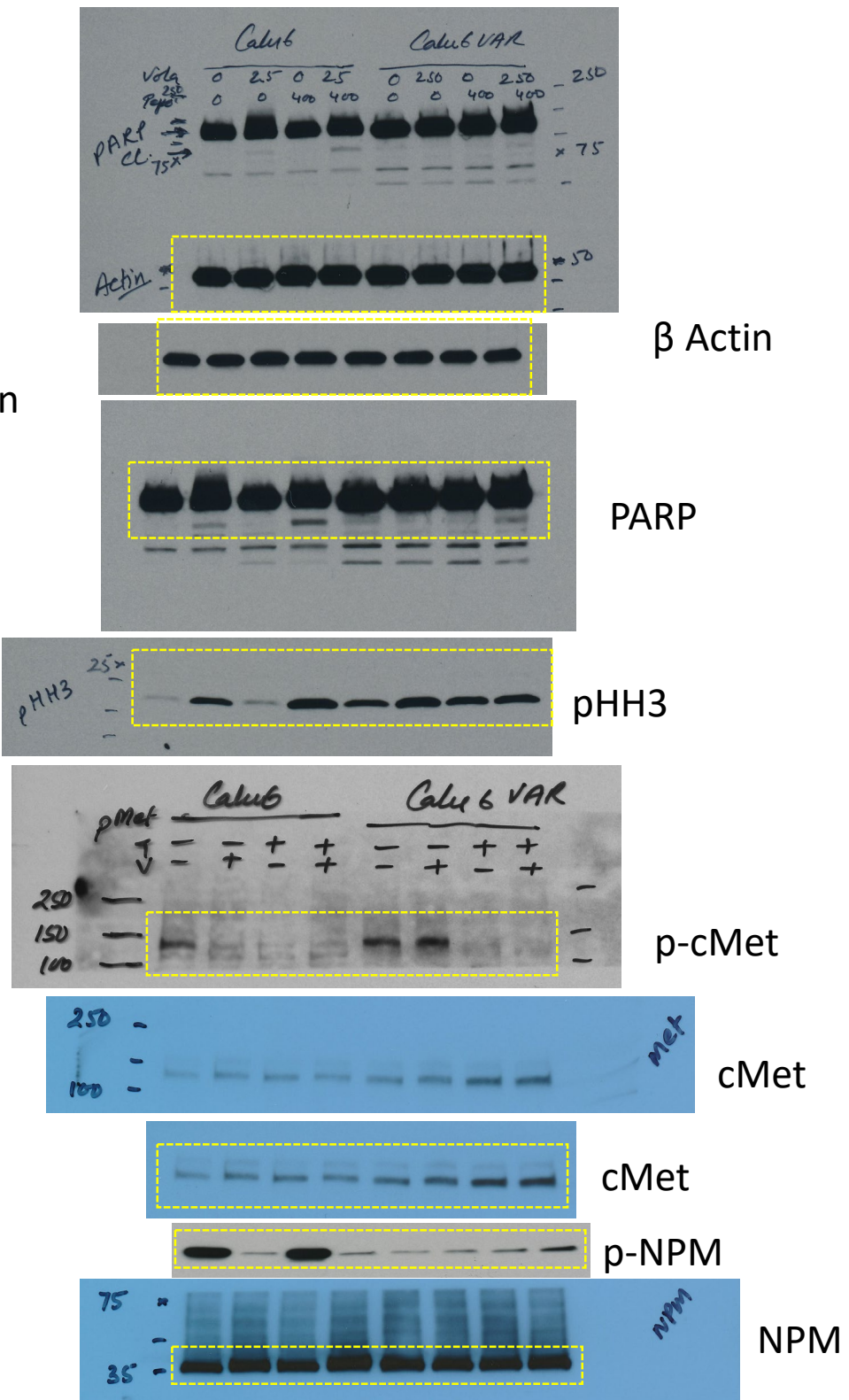

Supplement: Supplementary file 9 — Source Data for Figure 7 [file EMMM-11-e9960-s007.pdf]
