# Supplementary material for: Non‐canonical cMet regulation by vimentin mediates Plk1 inhibitor–induced apoptosis
Source: EMBO Mol Med. 2019 Apr 30;11(5):e9960. doi: 10.15252/emmm.201809960 (PMC6505578; doi:10.15252/emmm.201809960)

# Source data for Figure 3C

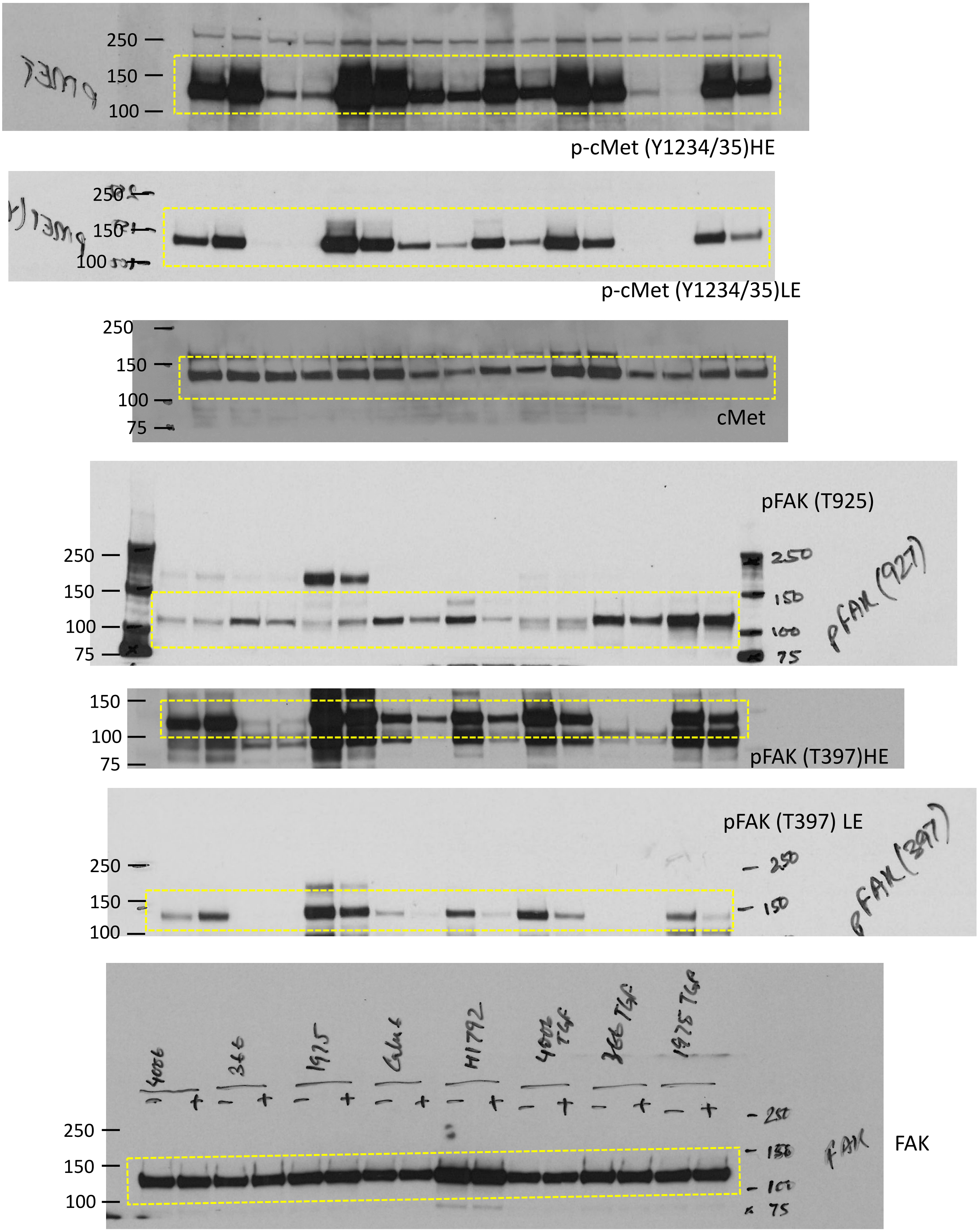

# Source data for Figure 3C

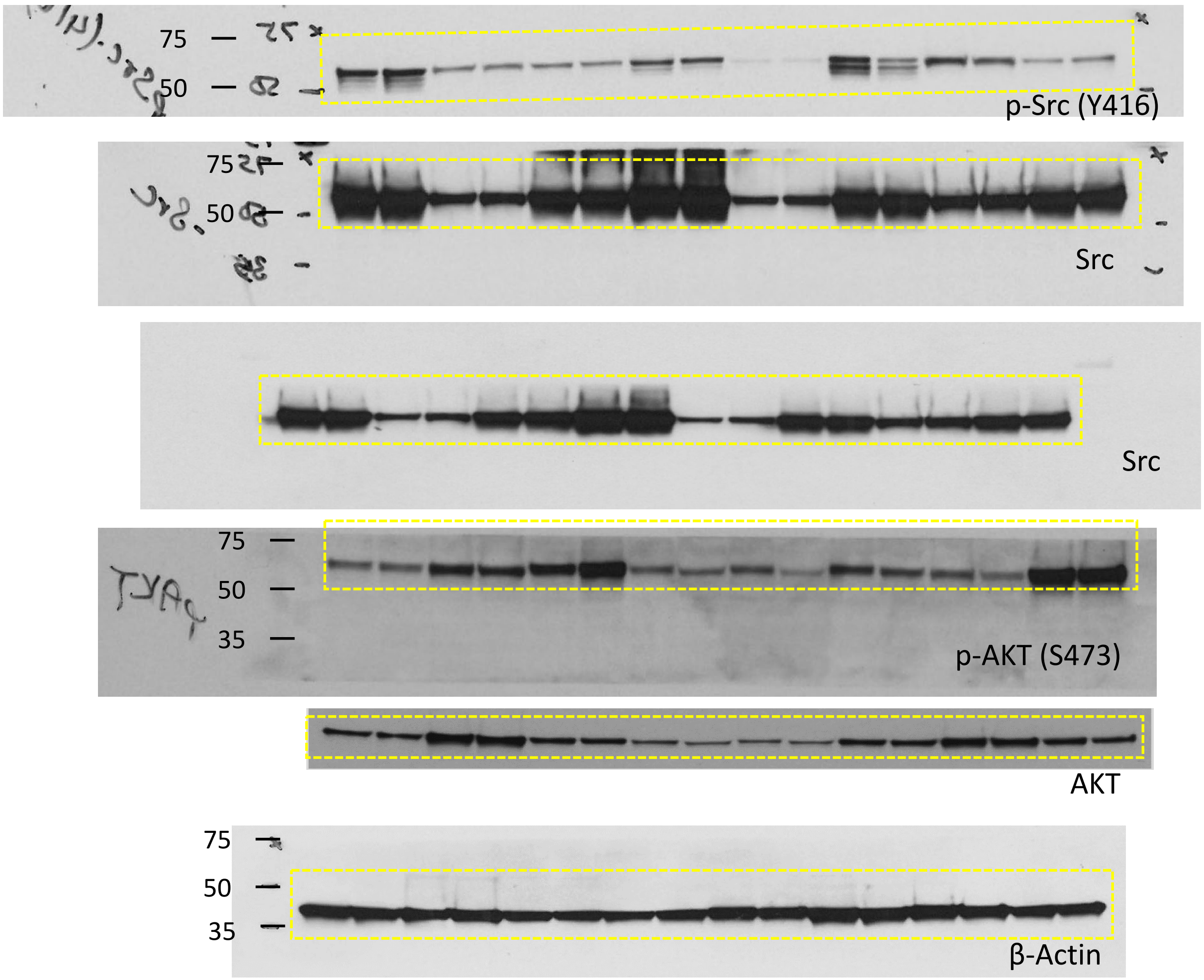

Source data for Figure 3D

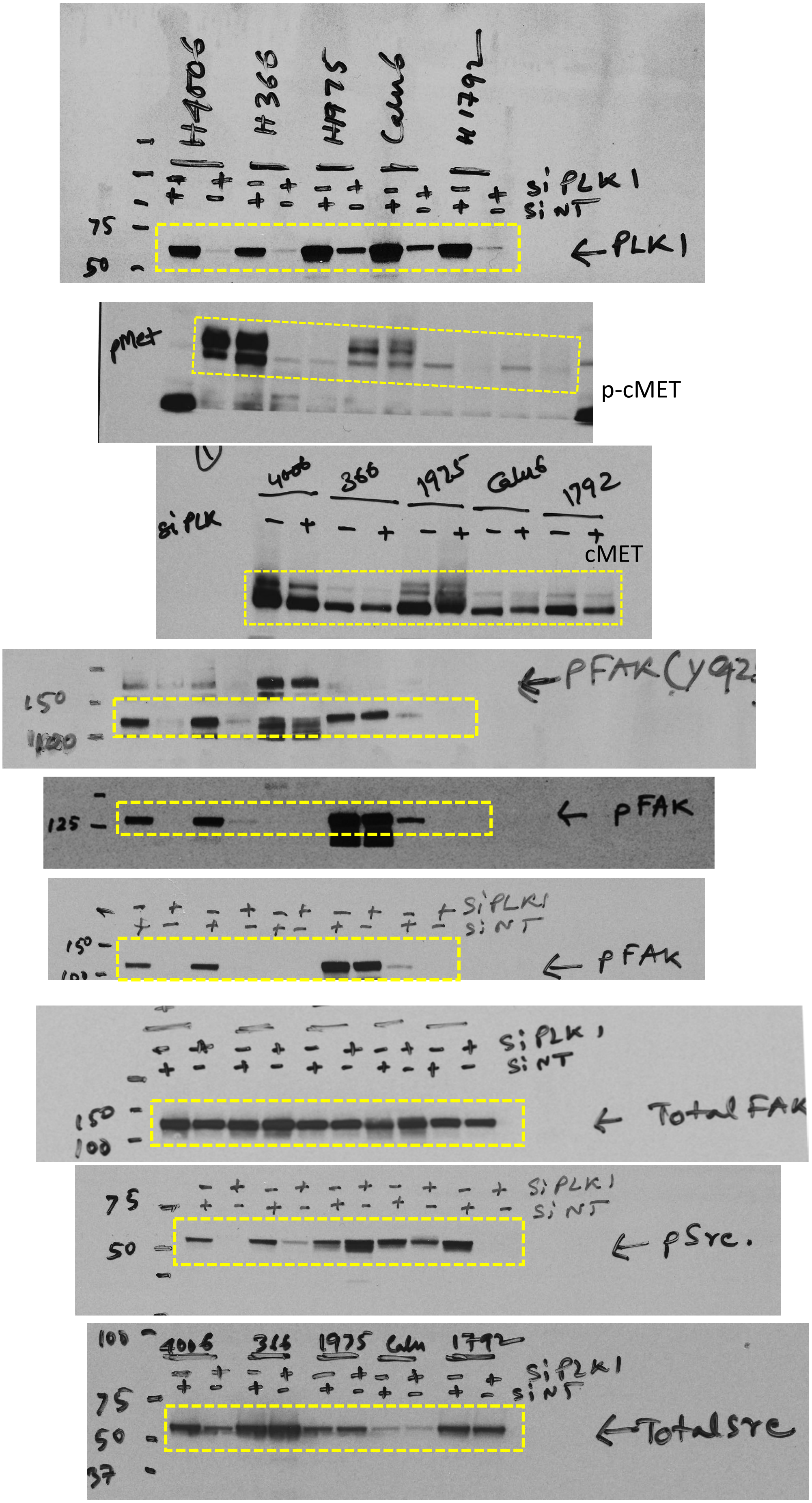

# Source data for Figure 3E

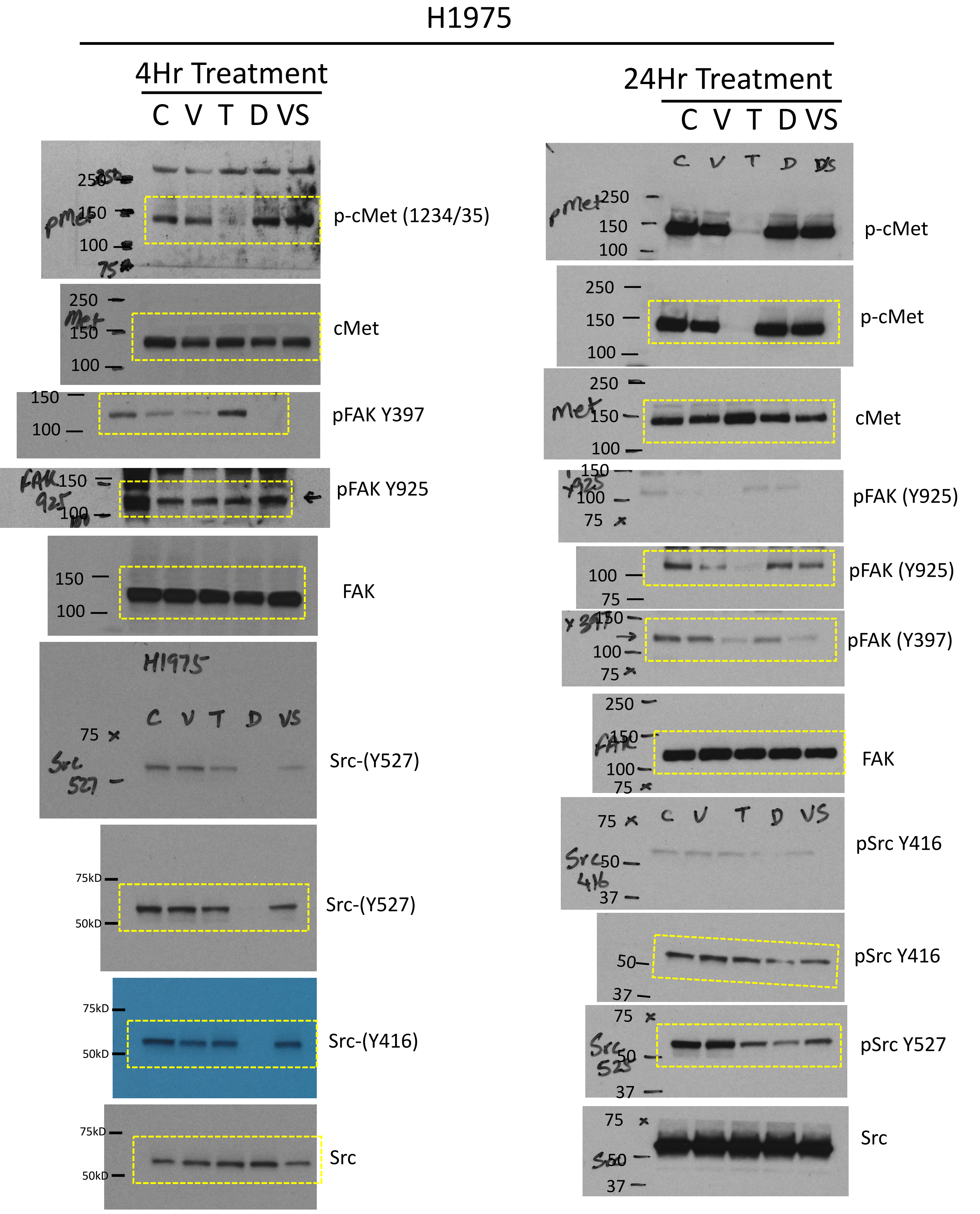

# Source data for Figure 3E

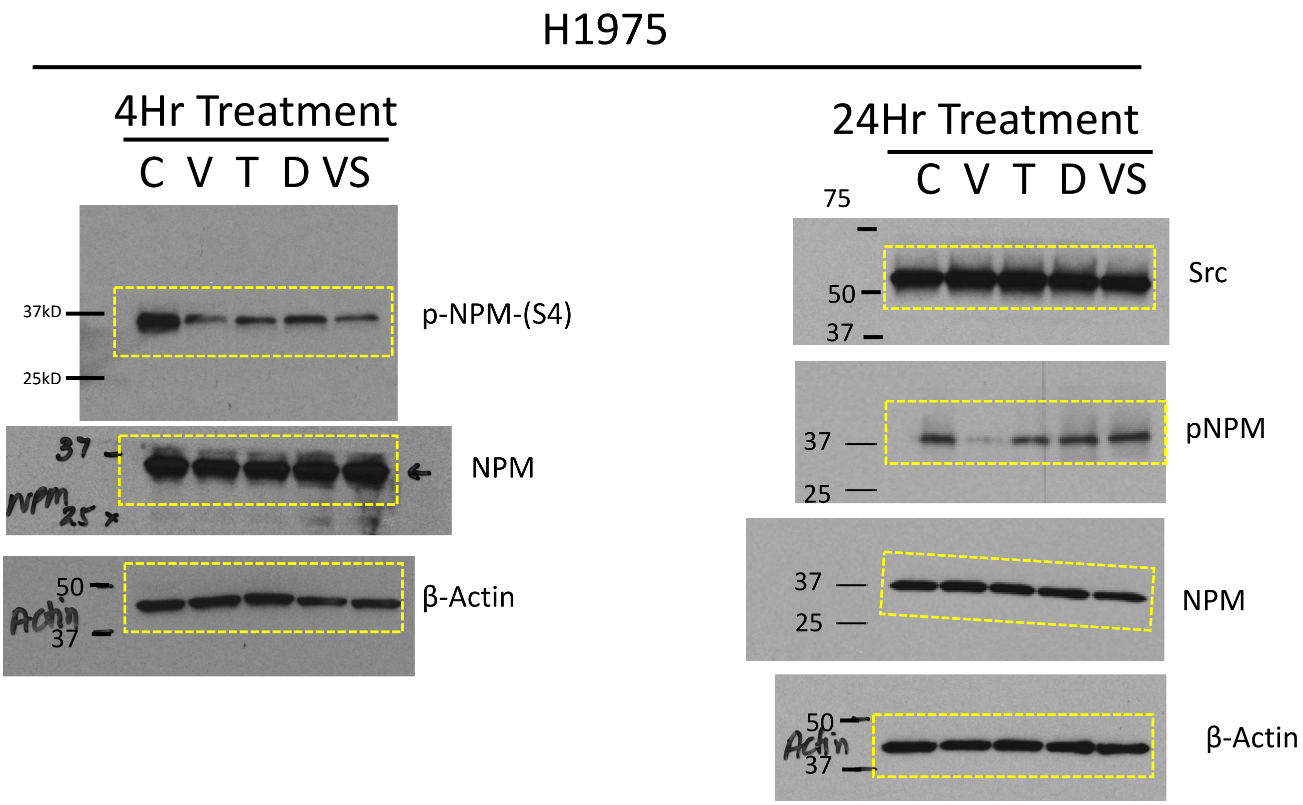

Source data for Figure 3E Calu6

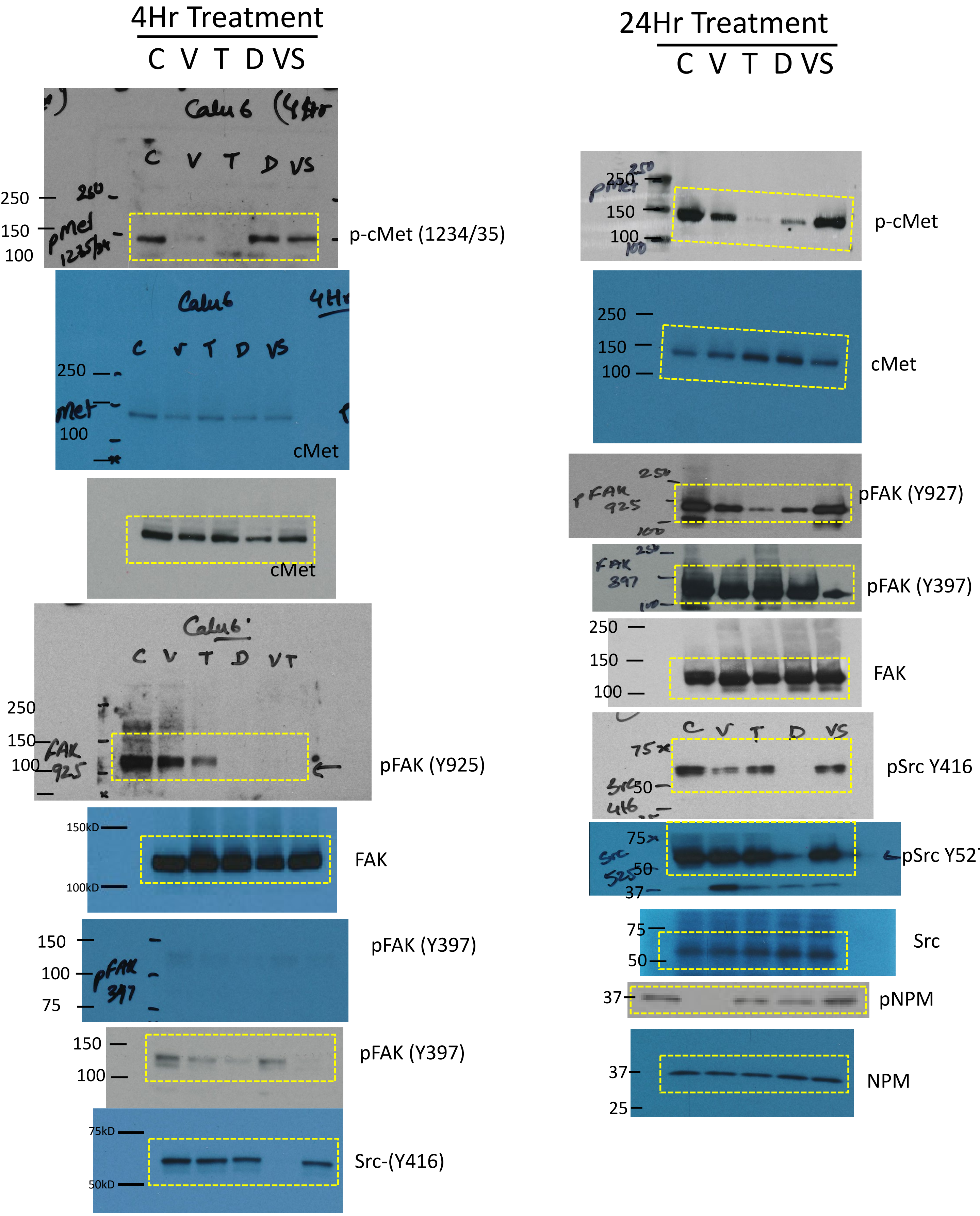

# Source data for Figure 3E

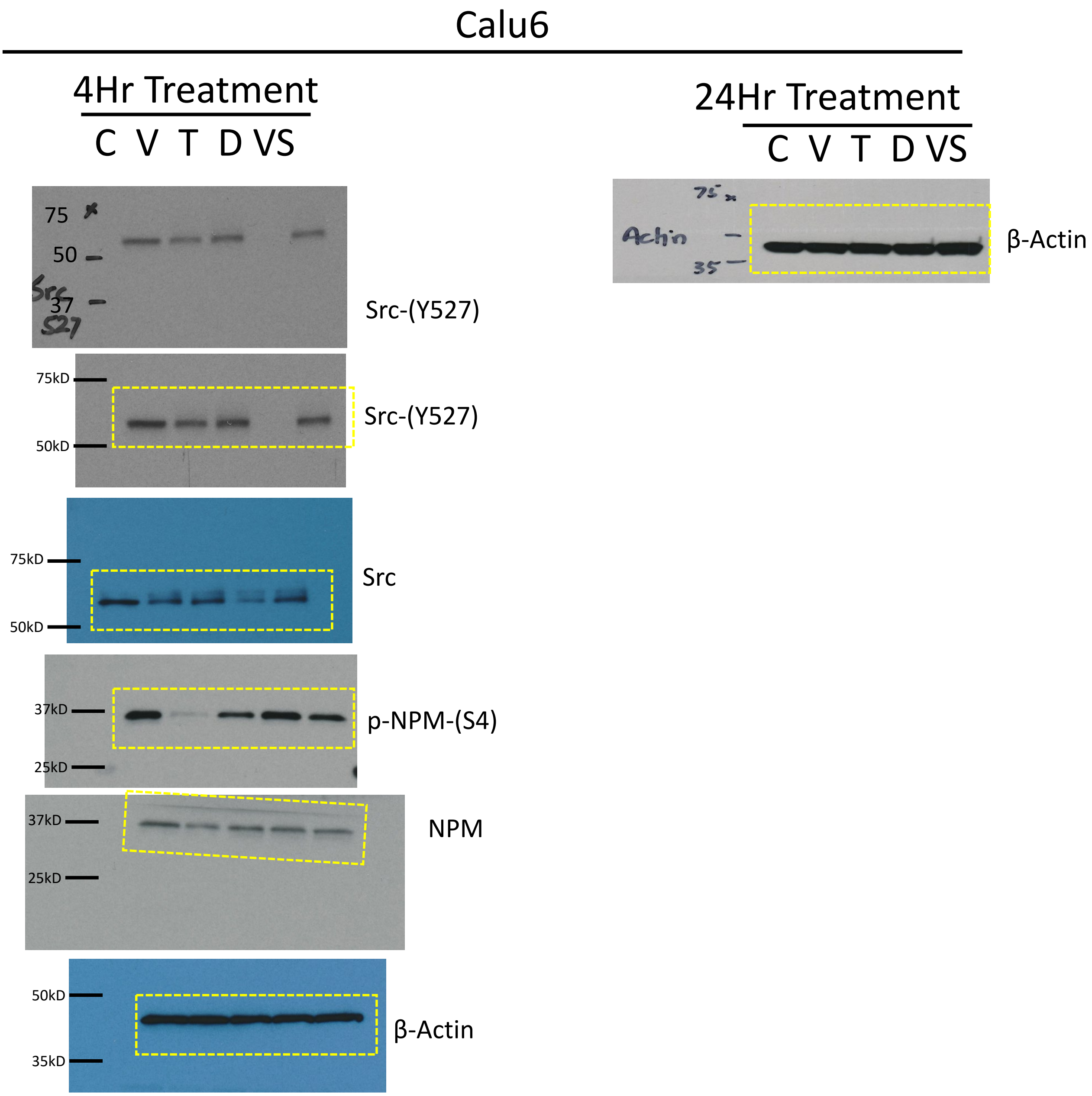

Supplement: Supplementary file 6 — Source Data for Figure 3 [file EMMM-11-e9960-s004.pdf]
