# Supplementary material for: Non‐canonical cMet regulation by vimentin mediates Plk1 inhibitor–induced apoptosis
Source: EMBO Mol Med. 2019 Apr 30;11(5):e9960. doi: 10.15252/emmm.201809960 (PMC6505578; doi:10.15252/emmm.201809960)

Source data for Figure 6B

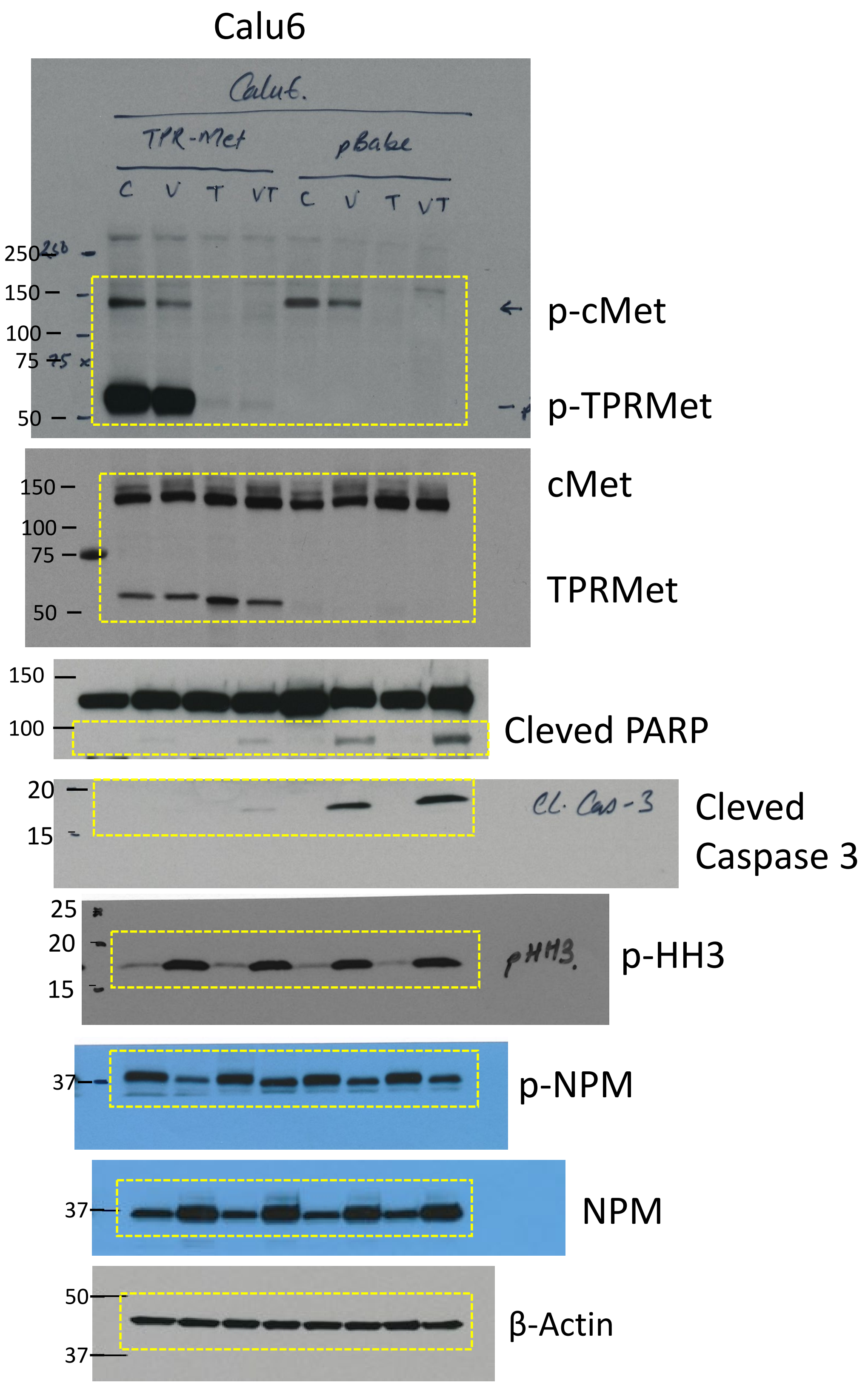

Source data for Figure 6B

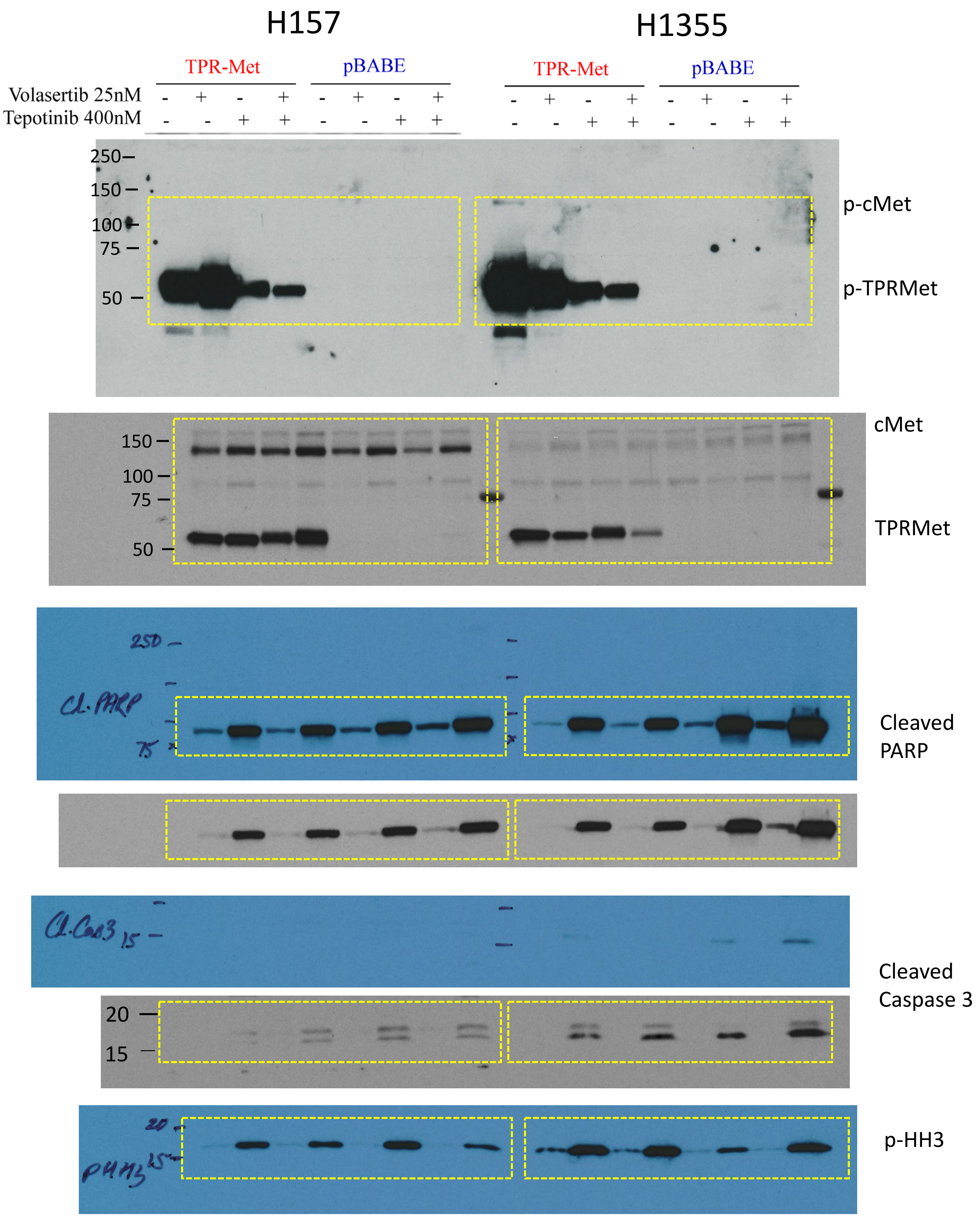

Source data for Figure 6B

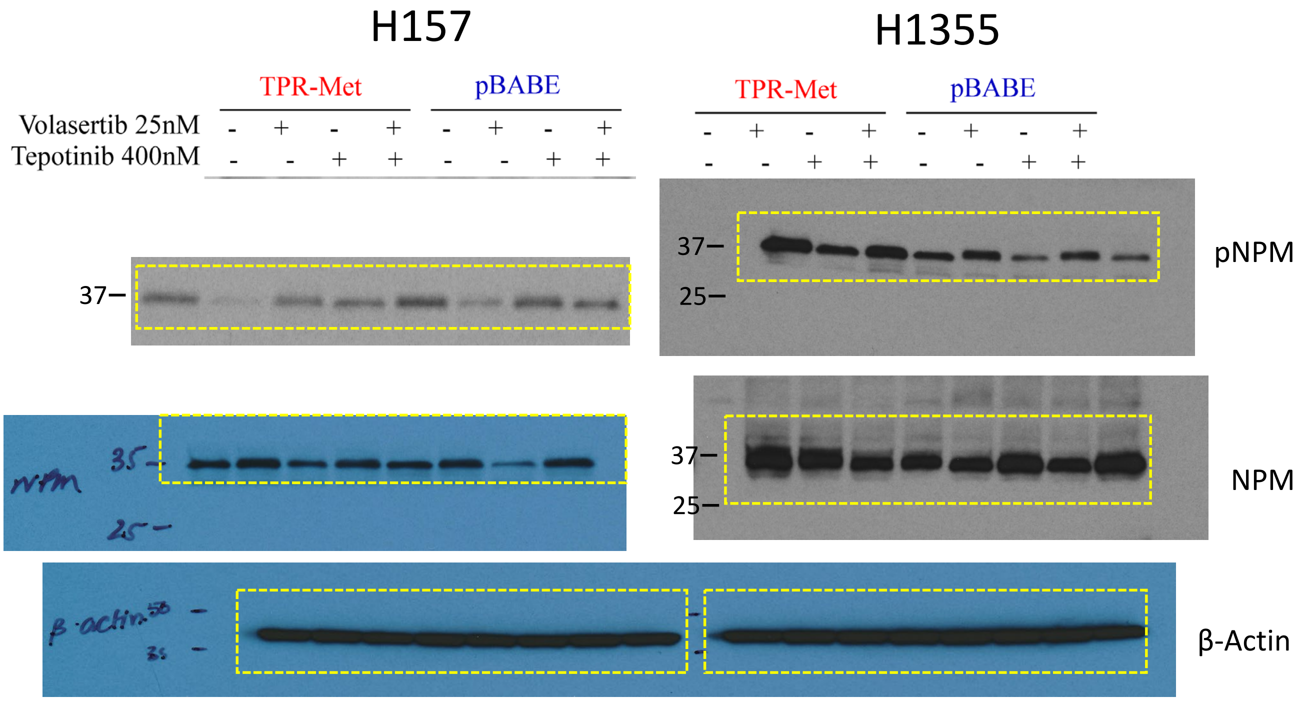

Source data for Figure 6D

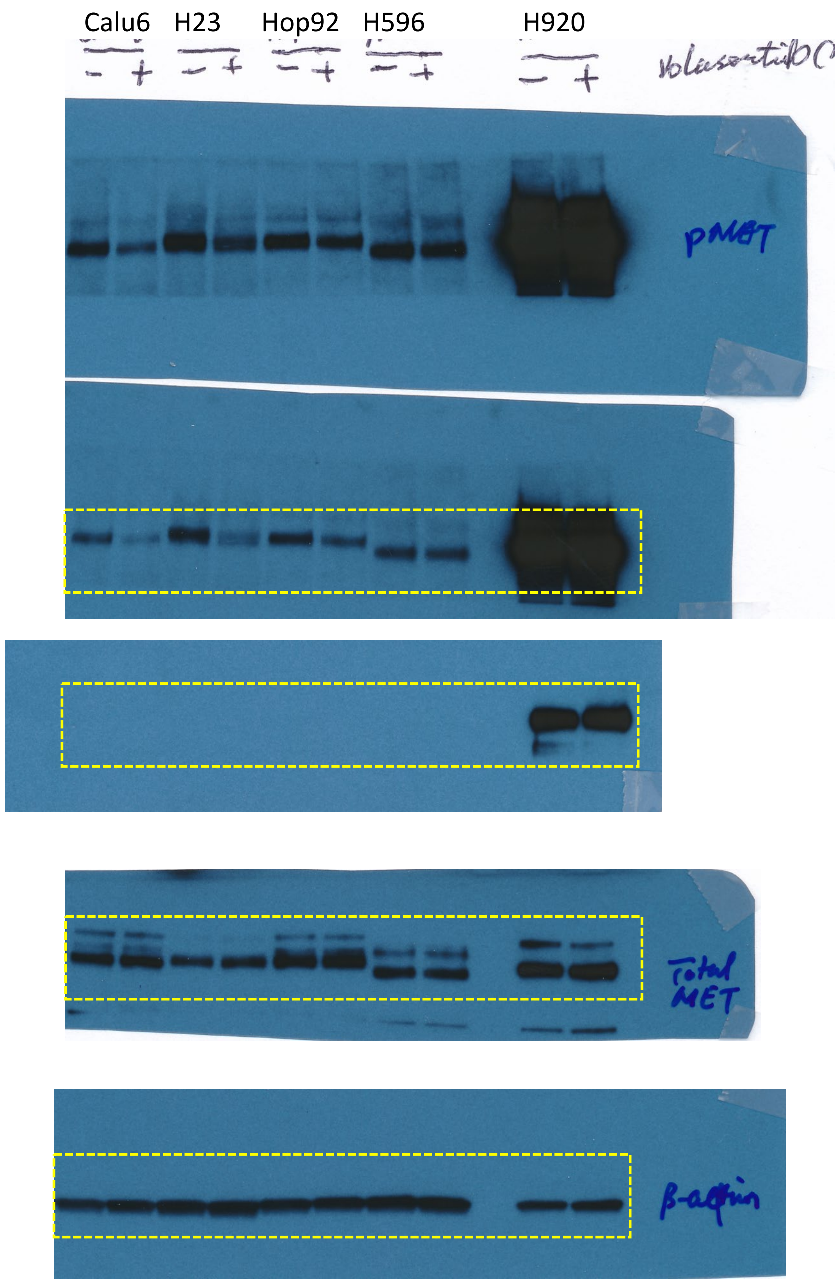

Supplement: Supplementary file 8 — Source Data for Figure 6 [file EMMM-11-e9960-s006.pdf]
