# Supplementary material for: Non‐canonical cMet regulation by vimentin mediates Plk1 inhibitor–induced apoptosis
Source: EMBO Mol Med. 2019 Apr 30;11(5):e9960. doi: 10.15252/emmm.201809960 (PMC6505578; doi:10.15252/emmm.201809960)

Source data for Figure 8A

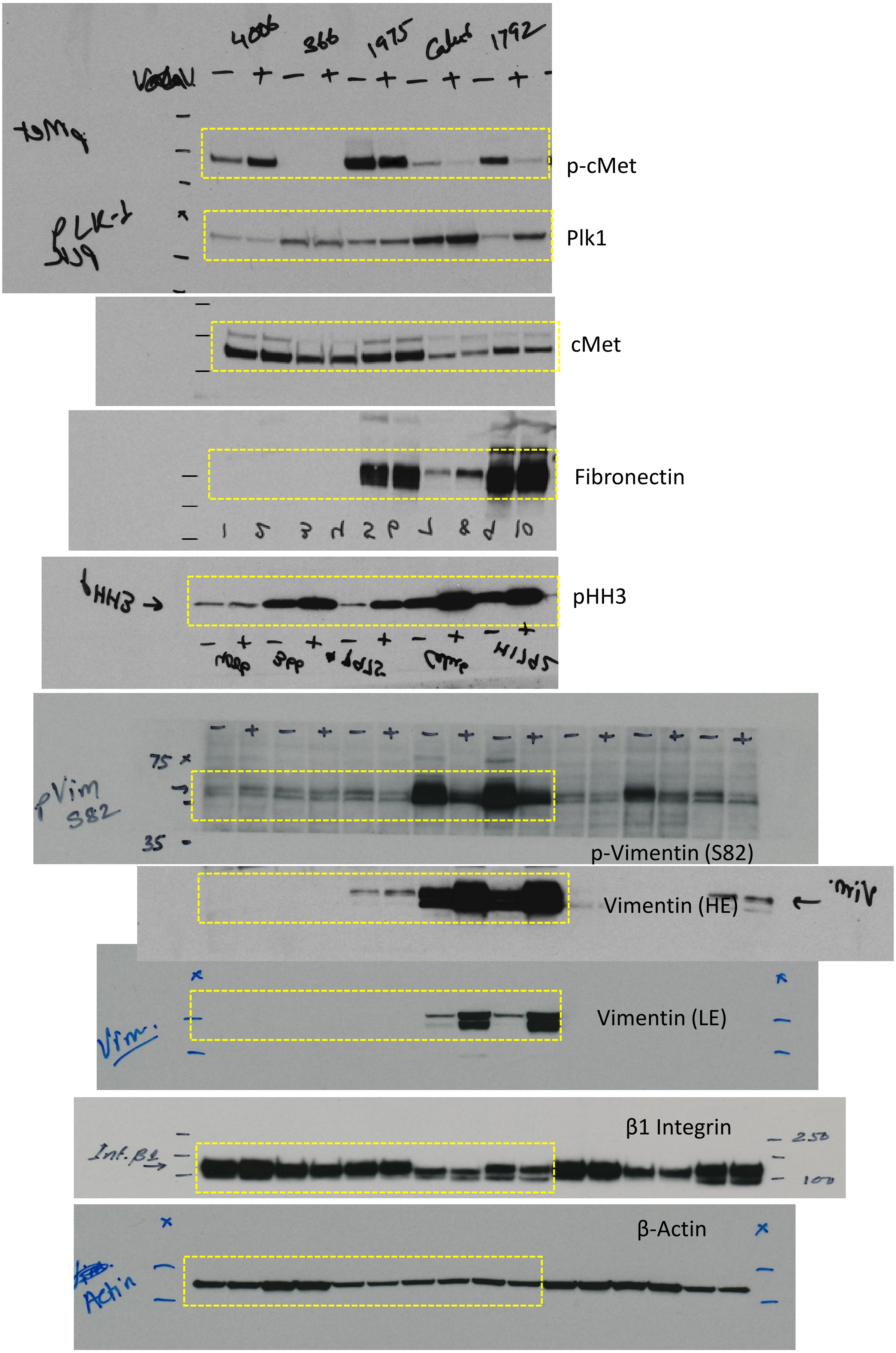

Source data for Figure 8B

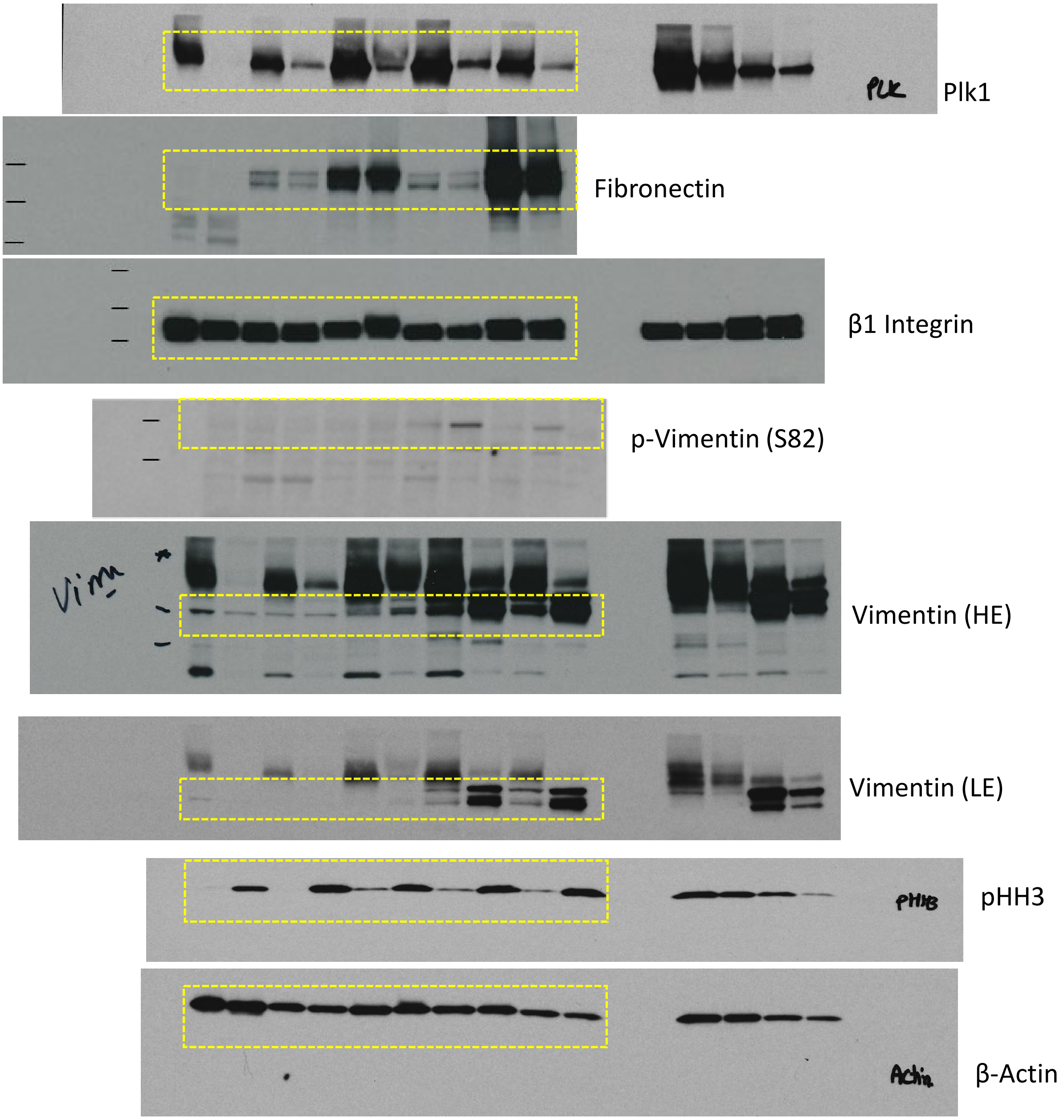

Source data for Figure 8C

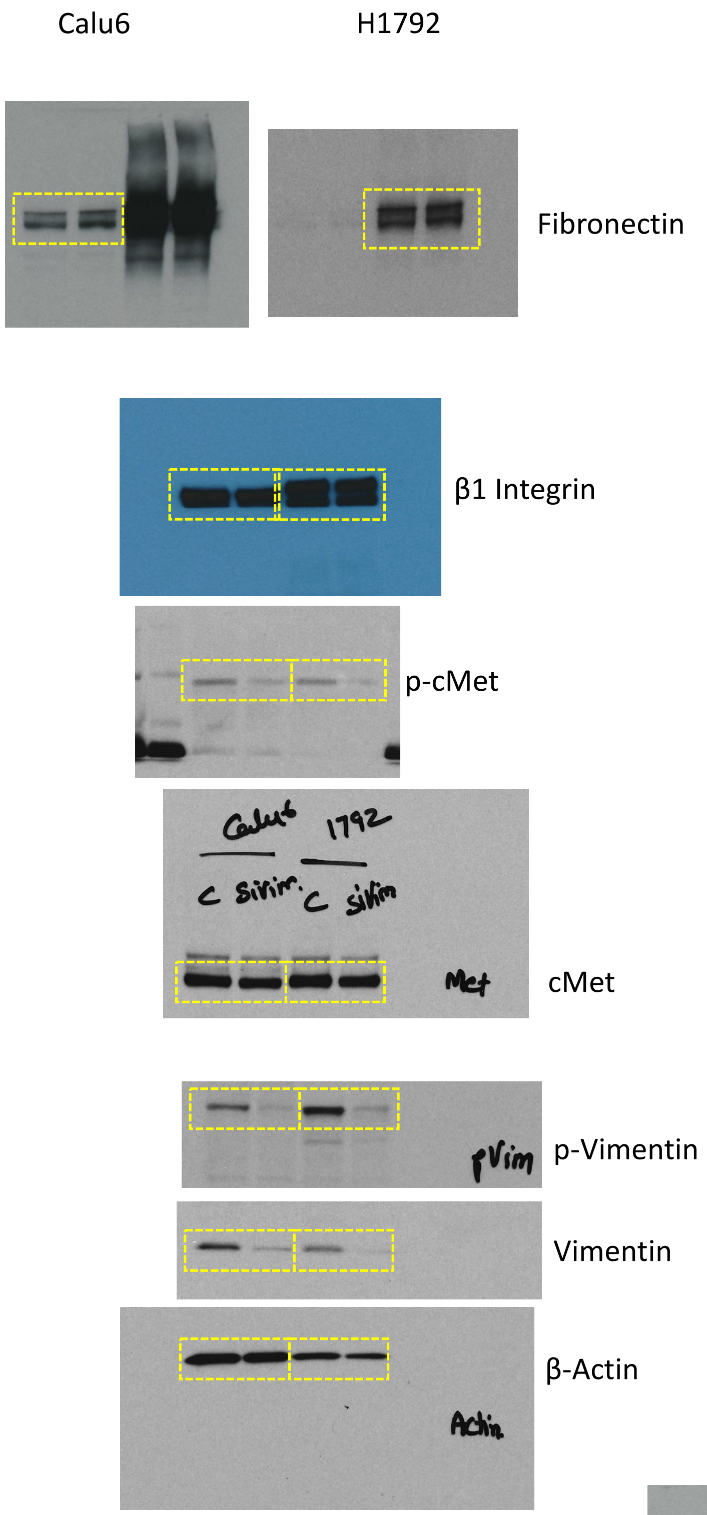

Source data for Figure 8D

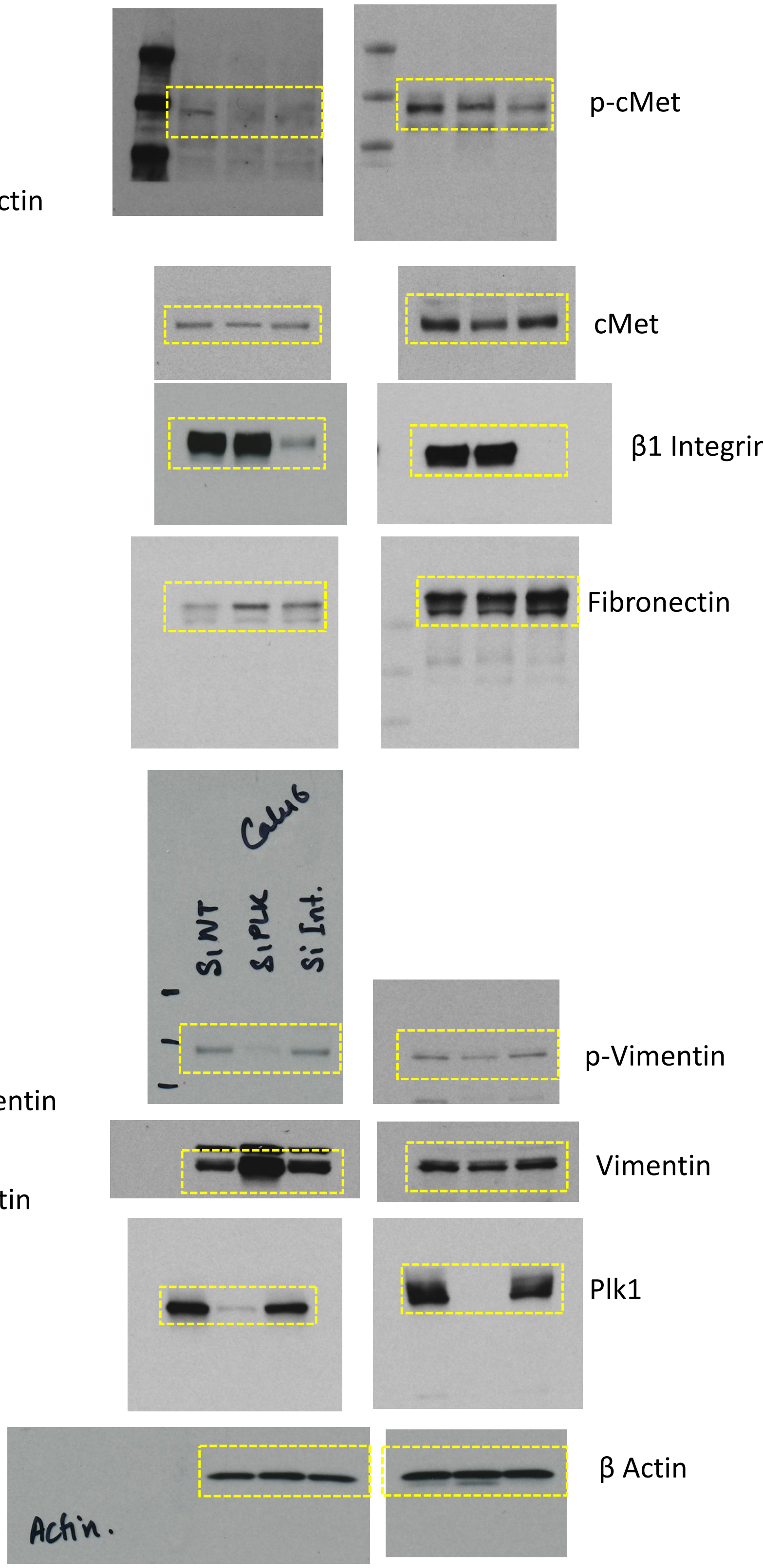

Source data for Figure 8E

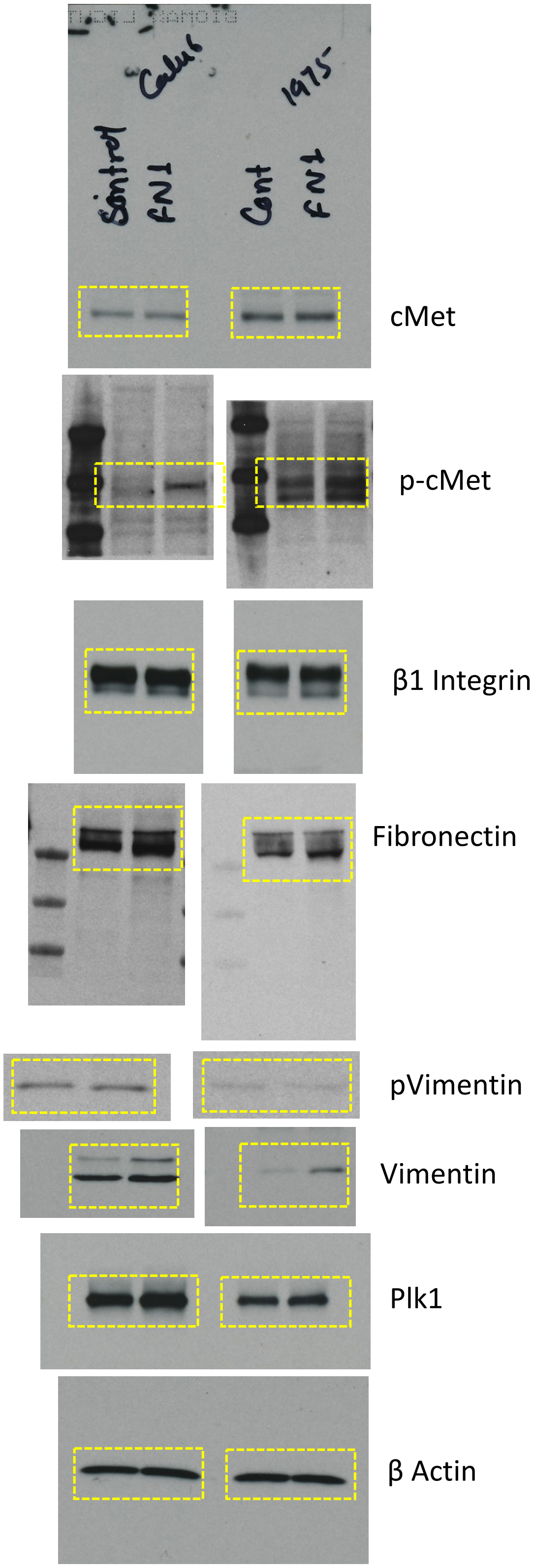

Source data for Figure 8F

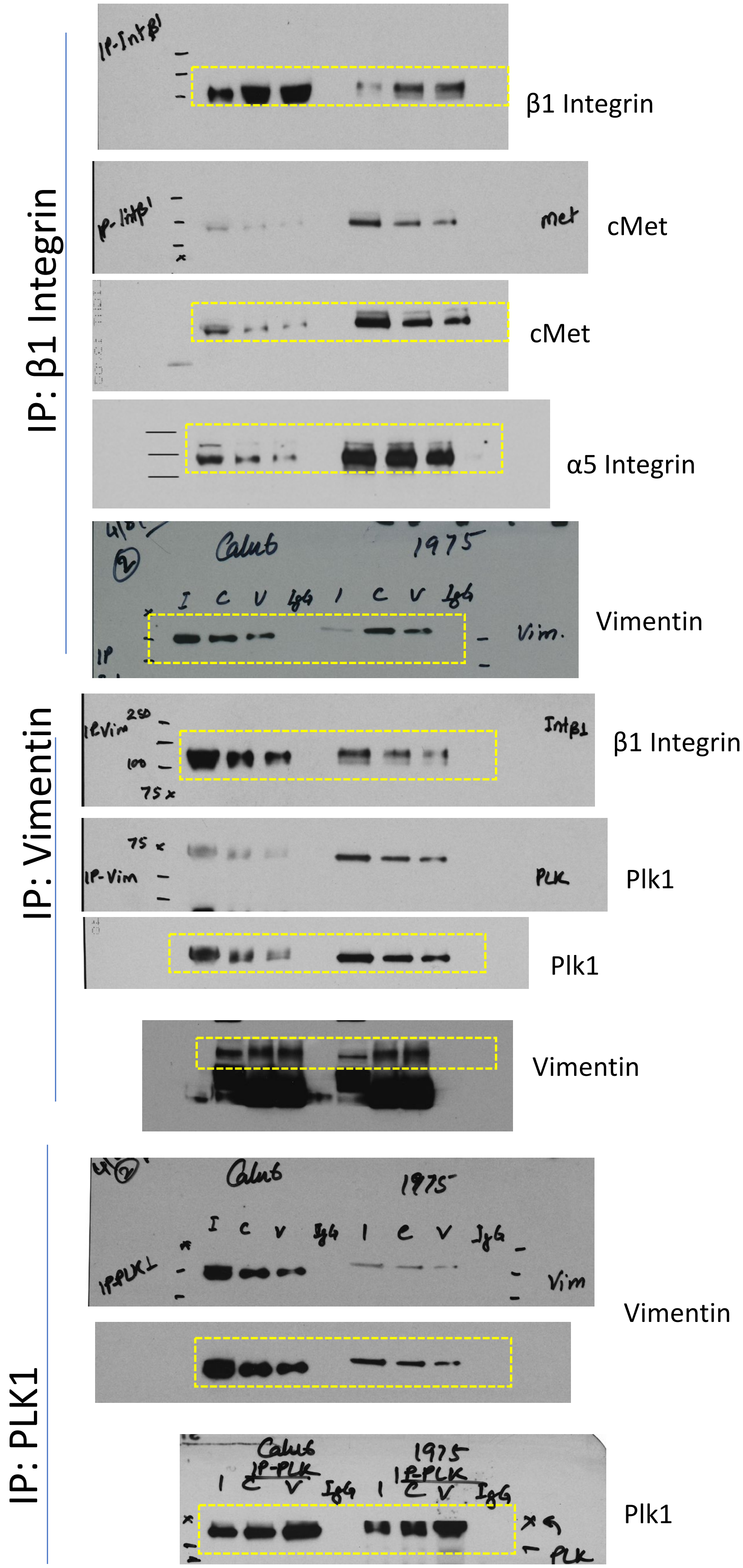

Supplement: Supplementary file 10 — Source Data for Figure 8 [file EMMM-11-e9960-s008.pdf]
